# Phthalimides: developments in synthesis and functionalization

**DOI:** 10.1039/d4ra03859b

**Published:** 2024-07-19

**Authors:** Fatemeh Doraghi, Mohammad Hossein Morshedsolouk, Nawrooz Ali Zahedi, Bagher Larijani, Mohammad Mahdavi

**Affiliations:** a Endocrinology and Metabolism Research Center, Endocrinology and Metabolism Clinical Sciences Institute, Tehran University of Medical Sciences Tehran Iran momahdavi@tums.ac.ir; b School of Chemistry, College of Science, University of Tehran Tehran Iran; c Department of Chemistry, Faculty of Education, Ghazni University Ghazni Afghanistan

## Abstract

Phthalimides, an important class of biologically active N-heterocycles, are not only found in pharmaceuticals, natural products, agrochemicals, polymers, and dyes, but also serve as building blocks in organic transformations. Many synthetic methods, including metal catalysis and metal-free systems, have been developed to prepare functionalized phthalimides. In this review, we describe the developments in the synthesis and functionalization of phthalimides over the past two decades.

## Abstract 2

Phthalimides are prevalent in pharmaceuticals, and natural products, *etc*. The synthesis of phthalimides catalyzed by transition metals and under metal-free conditions as well as functionalization of NH-phthalimides are described in this review.

## Graphical abstract

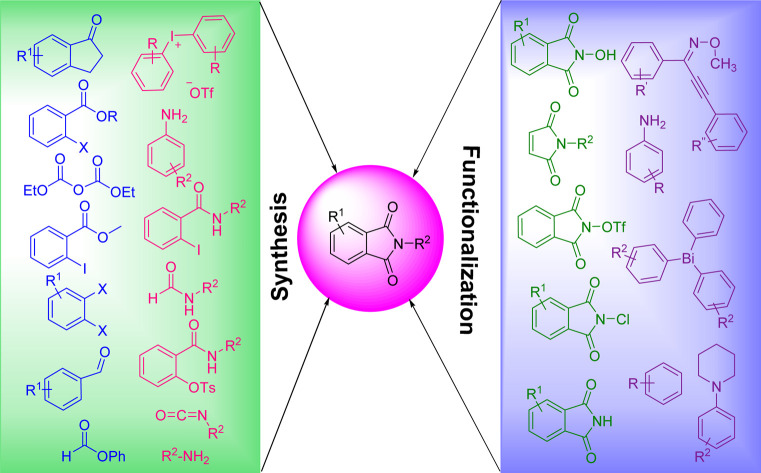

## 1. Introduction

**Scheme 1:**
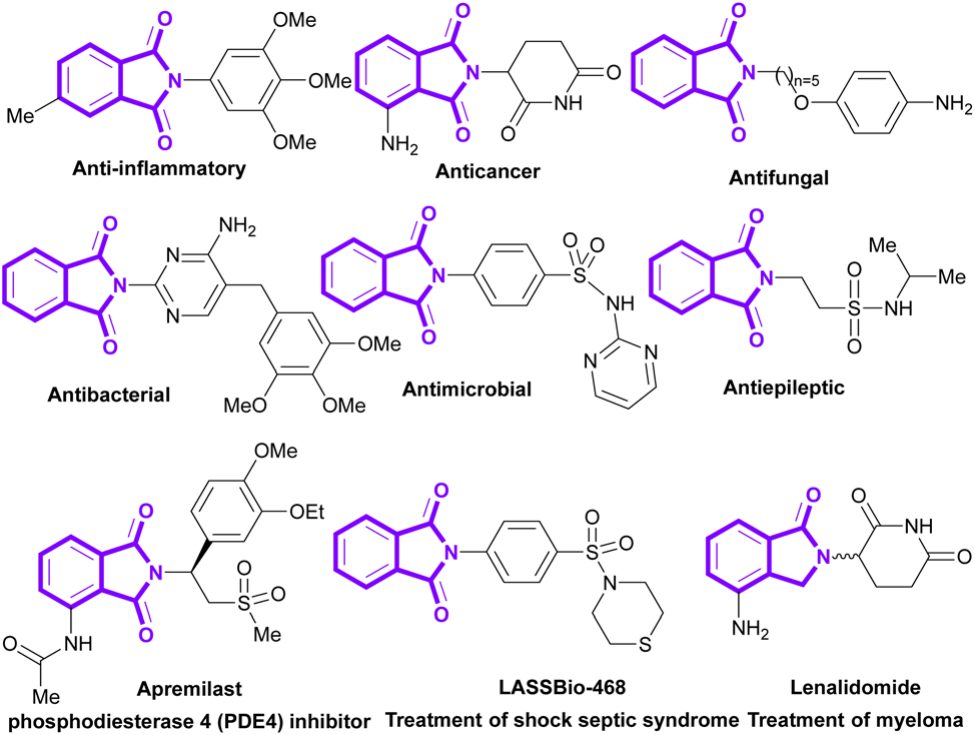
Representative examples of biologically active molecules containing phthalimide scaffold.

Phthalimides (isoindole-1,3-diones) are a well-known class of organic molecules, which are found in pharmaceuticals and natural products.^1–5^ They display abundant biological potentials, such as anticancer,^6–8^ antibacterial,^9,10^ anti-inflammatory,^11,12^ antimalarial,^13,14^ antifungal,^15^ anxiolytic,^16^ anti-HIV^17^ and anticonvulsant^18,19^ activities. Some phthalimide derivatives are in the drug market to treat psoriasis (apremilast),^20^ myeloma (lenalidomide),^21^ rheumatoid arthritis,^22^ and shock septic syndrome (LASSBio-468).^23^ In addition to numerous medicinal applications, phthalimides show good potential in agrochemical, polymer and dye industries. Recently, the use of phthalimides was reported in the preparation of pesticides,^24^ rubber^25^ and dyestuff.^26^ Some important bioactive derivatives of phthalimides are outlined in Scheme 1.

Thus, the synthesis and functionalization of these valuable frameworks is highly important. Since the first application of phthalimide in organic synthesis by S. Gabriel in 1887,^27^ the use of phthalimide derivatives as versatile precursors in organic transformations have achieved great interest.^28,29^ Various synthetic methods have been documented for the preparation of phthalimides, including metal-catalyzed aminocarbonylation cyclizations of *ortho*-dihaloarenes or *ortho*-haloarenes, the amidation of phthalic acid/anhydride by primary amines and annulations involving maleimide. However, in many of these synthetic strategies, CO gas is used as a carbonyl precursor, and due to its many disadvantages, such as toxicity, lack of odor; flammability and issues related to storage, handling, transportation and safety, research efforts are undertaken to search for less toxic, more accessible and easy to handle carbonyl precursors.

In addition to the preparation of these scaffolds, the functionalization of phthalimides, especially *N*-arylation, has received widespread attention.^30^ In this context, recently, remarkable approaches to the functionalization reactions of phthalimides through C–N bond formation *via* C(sp^2^)–H/C(sp^3^)–H bond cleavage have been developed under metal or non-metal catalysis.

Owing to the high importance of phthalimide derivatives, in the current review, we describe the development in the synthesis of phthalimides and *N*-functionalizations of these scaffolds. In this regard, the review is divided into two main categories: the synthesis and the functionalization of phthalimides. The first section is classified on the basis of the transition metal catalyzed reaction and metal-free synthetic method, and the next part is devoted to the *N*-arylation and *N*-alkenylation of phthalimides. Furthermore, the scope of substrates and important reaction mechanisms are discussed.

## 2. Synthesis of phthalimides

### 2.1. Transition metal-catalyzed synthesis of phthalimides

#### 2.1.1. Ni-catalyzed synthesis of phthalimides

**Scheme 2:**
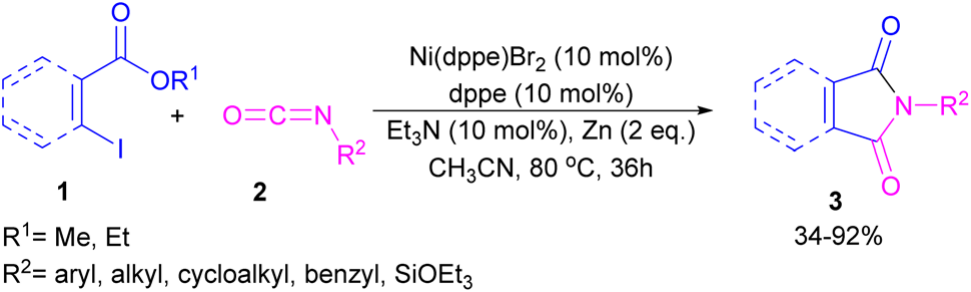
Ni-catalyzed coupling of isocyanates with 1,3-iodoesters and halobenzenes.

**Scheme 3:**
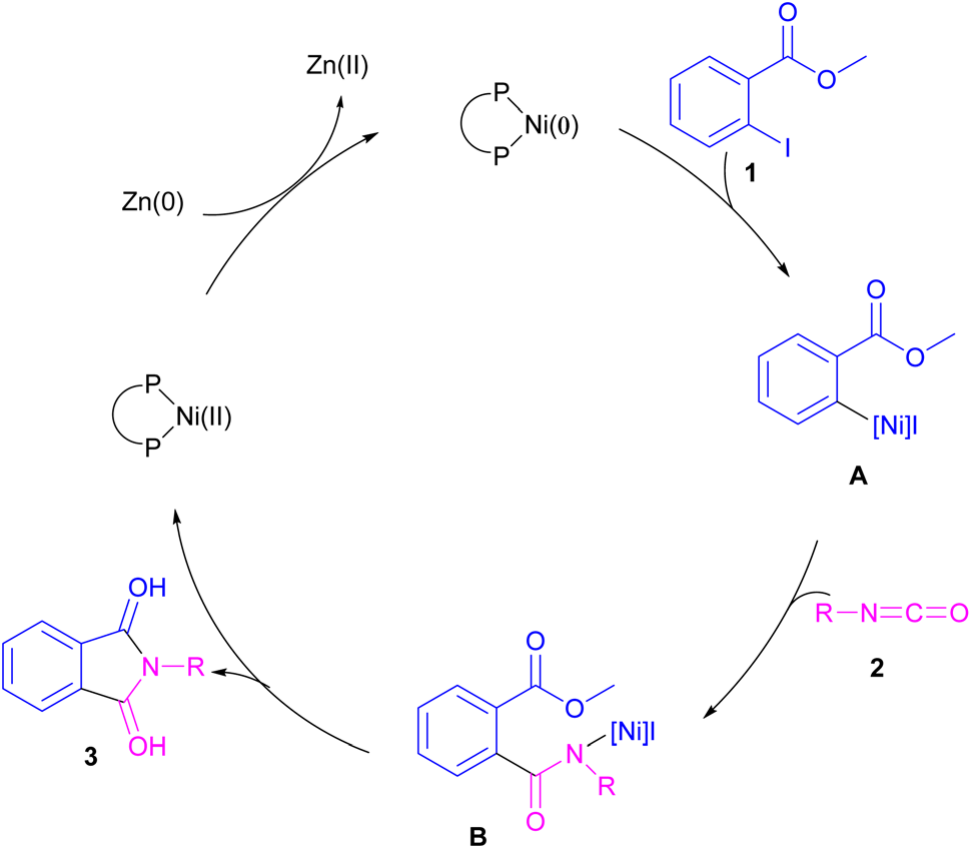
Possible catalytic cycle for Ni-catalyzed coupling of isocyanates with 1,3-iodoesters and halobenzenes.

The first example of coupling of aliphatic and aromatic isocyanates (2) with 1,3-iodoesters and *ortho*-iodobenzenes (1) catalyzed by a nickel catalyst was reported by Cheng and co-workers in 2005 (Scheme 2).^31^ The reaction was influenced by the electronic nature of the functional groups on the aryl ring of isocyanate, where an electron-donating substituent enhanced the reactivity but an electron-withdrawing substituent did not. Moreover, alkylisocyanates resulted in higher yields compared to arylisocyanates. In addition, a siloxane group on isocyanate could give a 92% yield of the product. As shown in Scheme 3, the reaction involved the reduction of Ni(ii) to Ni(0) by zinc powder to insert into the C–I bond through oxidative addition. The obtained intermediate A then underwent the isocyanate insertion to form intermediate B. Intramolecular imidation furnished product 3 along with the release of an ester group and the regeneration of Ni(ii).

#### 2.1.2. Cu-catalyzed synthesis of phthalimides

**Scheme 4:**
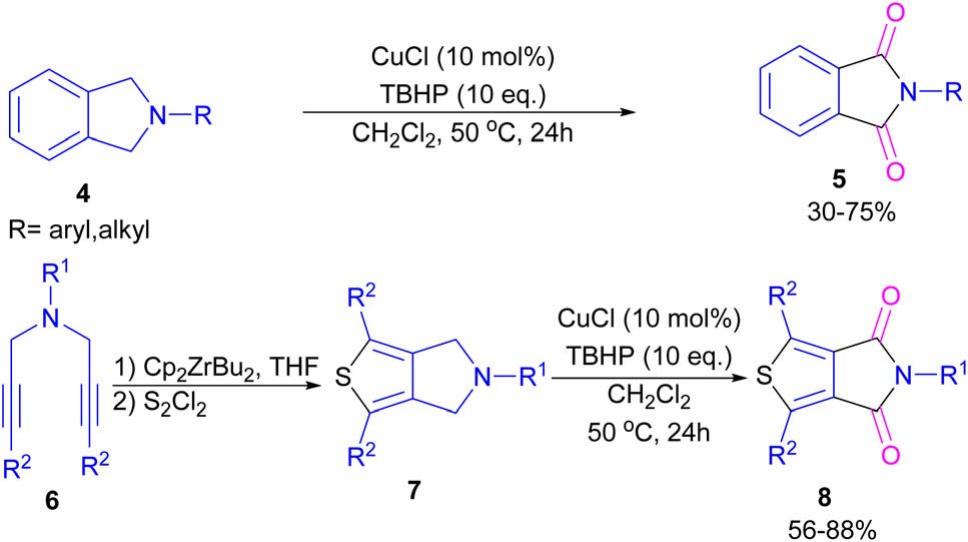
Cu/TBHP-catalyzed oxidation of arene-fused cyclic amines.

Xie and co-workers developed an efficient Cu/TBHP catalysis system for the construction of phthalimide derivatives 5 through the oxidation of arene-fused cyclic amines 4 (Scheme 4).^32^ Various Cu catalysts, such as Cu(OAc)_2_, CuCl_2_, CuCl, CuBr, and CuI led to phthalimide products in good yields and among them, the best result was obtained using CuCl as a catalyst. This synthetic method was also used for the synthesis of a building block in organic solar cells and organic field-effect transistors, namely thieno[3,4-*c*]pyrrole-4,6-dione (TPD) 5. This reaction involved the cyclization of bispropargylamines 6 to 6-dihydrothieno[3,4-*c*]pyrroles 7 in the presence of zirconocene, followed by the oxidation under the Cu/TBHP catalysis system to form TPD 8.

**Scheme 5:**
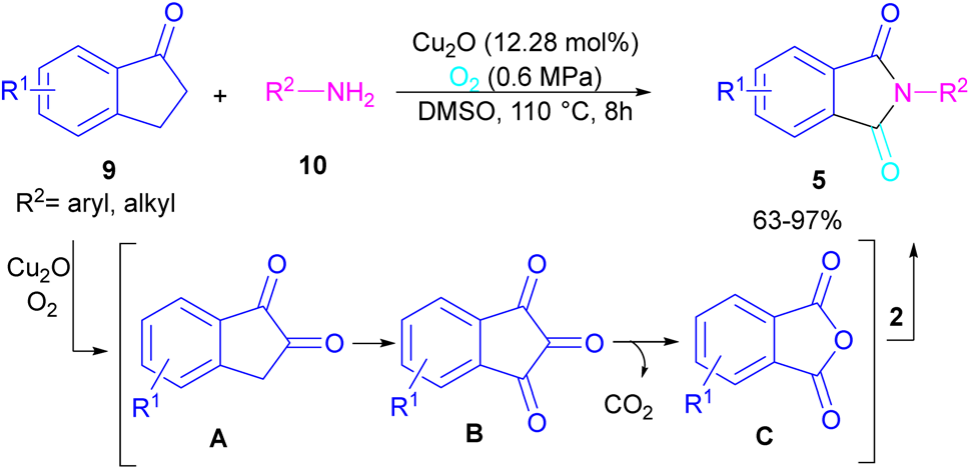
Cu-catalyzed oxidative reaction of ketones and amines.

*N*-Substituted phthalimides can be synthesized from 1-indanones 9 and aryl/alkyl amines 10 in the presence of CuO_2_ as a catalyst (Scheme 5).^33^ The reaction involved the C–C bond cleavage and the C–N bond formation access to phthalimide, where O_2_ acted as a green oxygen source. DFT calculations revealed the possibility of both α-C–H as well as β-C–H activation in the reaction. The formation of 1,2-indandione A occurred under Cu catalysis in the presence of O_2_, which by further oxidation gave 1,2,3-indantrione B. The extrusion of CO_2_ from B led to *ortho*-phthalic anhydride, which reacted with amine 10 to form imide 5.

**Scheme 6:**
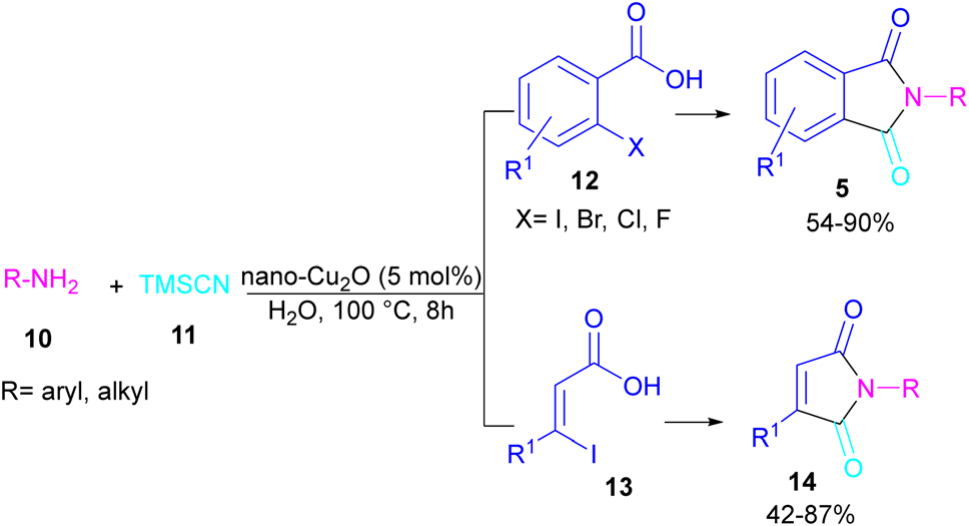
Cu-cyanation–annulation–aminolysis reaction toward phthalimides.

**Scheme 7:**
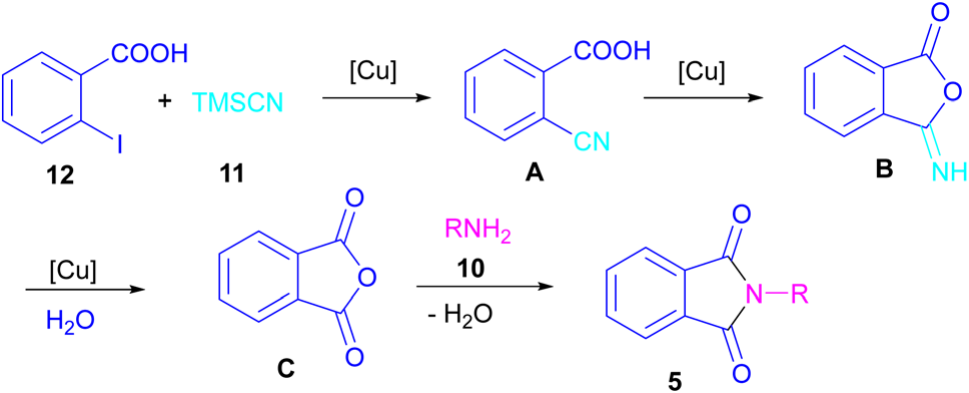
Proposed mechanism for Cu-cyanation–annulation–aminolysis reaction toward phthalimides.

In 2021, a nano-Cu_2_O catalyst was employed by Chen *et al.* for the assembly of phthalimides (Scheme 6).^34^ A wide variety of amines 10, 2-halobenzoic acids 12 and TMSCN 11 were treated in the presence of a nanocatalyst in water as a solvent to obtain the *N*-substituted phthalimides 5. In addition to phthalimides, a series of malimides 14 was also constructed in this method through the cyclization of β-iodoacrylic acids 13. It was found that Cu(i) nanocatalyst is involved in all reaction steps, including cyanation, cyclization and hydrolysis. The mechanistic investigations indicated that the two intermediates A and C are involved and TMSCN is necessary in the reaction. The reaction mechanism started with the generation of 2-cyanobenzoic acid A*via* the cyanation of 2-iodobenzoic acid 12 with TMSCN 11 under copper catalysis, followed by an intramolecular nucleophilic addition toward intermediate B. After that, the hydrolysis of B to phthalic anhydride C, and subsequent amine attack led to product 5 along with the elimination of a H_2_O molecule (Scheme 7). This strategy has the advantages of low catalytic loading, the use of green solvent H_2_O, and no need for CO, ligand, or an additive in the reaction.

**Scheme 8:**
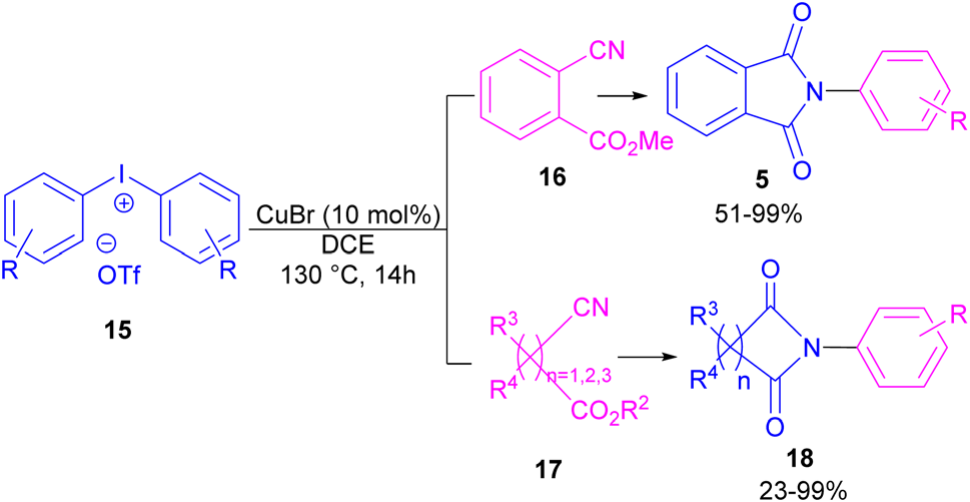
Cu-catalyzed reaction of cyanoesters with diaryliodonium salts.

**Scheme 9:**
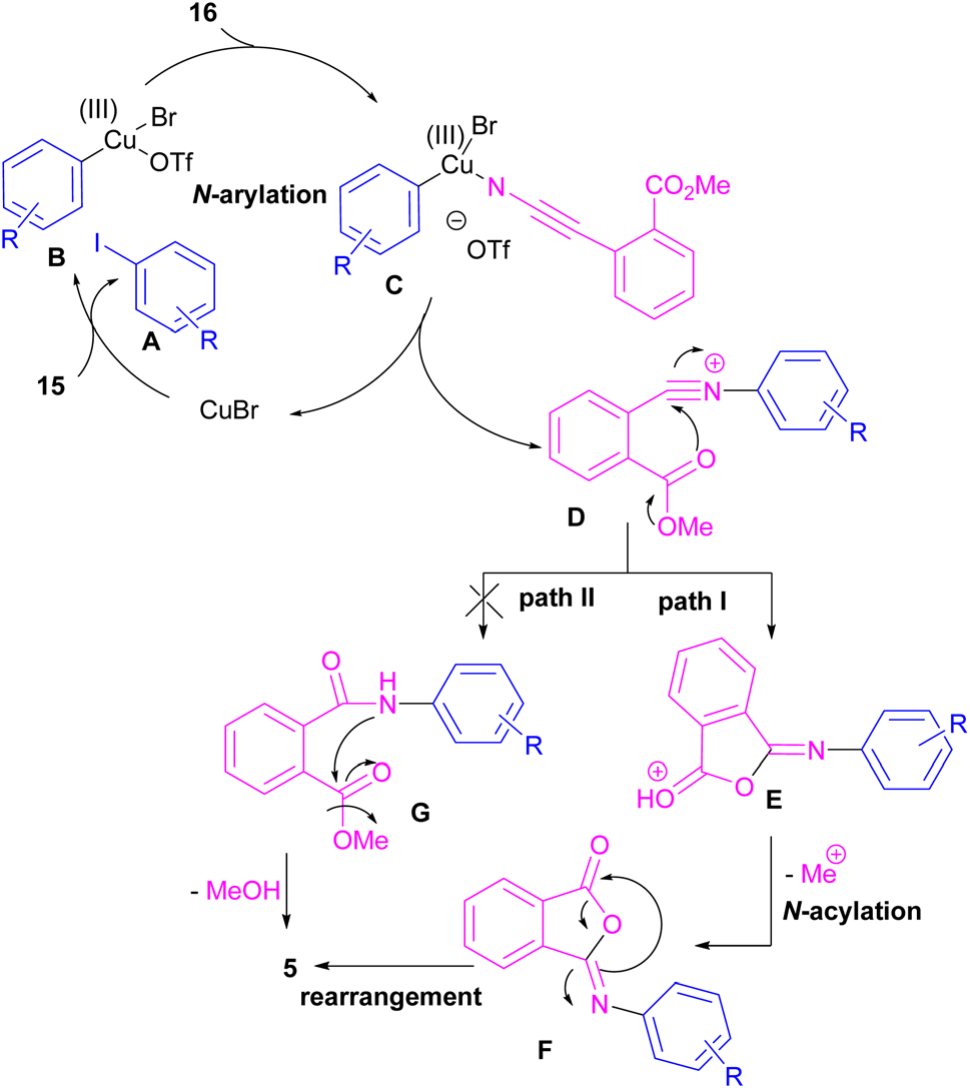
Possible mechanism for Cu-catalyzed reaction of cyanoesters with diaryliodonium salts.

Chen and Bao prepared phthalimides 5 and unsubstituted cyclic imides 18 from copper-catalyzed cyclization of diaryliodonium salts 15 with cyanoesters 16 or 17 (Scheme 8).^35^ In general, the reaction involved the initial interaction between CuBr and diaryliodonium salts 15 to form Cu(iii) complex B and aryl iodide A. The coordination of cyanoester 16 to the Cu center resulted in intermediate C, which was further converted to a more reactive intermediate D through the elimination of Cu(i). This intermediate can proceed in two possible pathways. In path I, an intramolecular nucleophilic addition led to intermediate E, followed by the N-acylation to yield the isophthalimide F. Finally, phthalimide 5 was furnished *via* a 1,3-(O–N) acyl transfer rearrangement process. Another possible pathway that can be considered for this transformation is *via* the amide formation (intermediate G in path II). However, with a control experiment that treated substrate 15 with an amide reagent, no phthalimide product was formed, ruling out this pathway (Scheme 9).

#### 2.1.3. Co-catalyzed synthesis of phthalimides

**Scheme 10:**
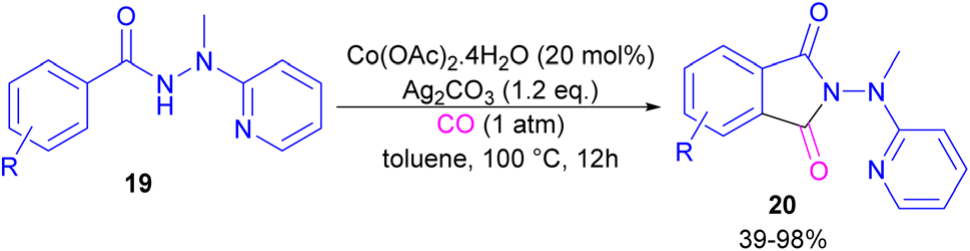
Co-catalyzed carbonylation of benzoyl hydrazide derivatives.

**Scheme 11:**
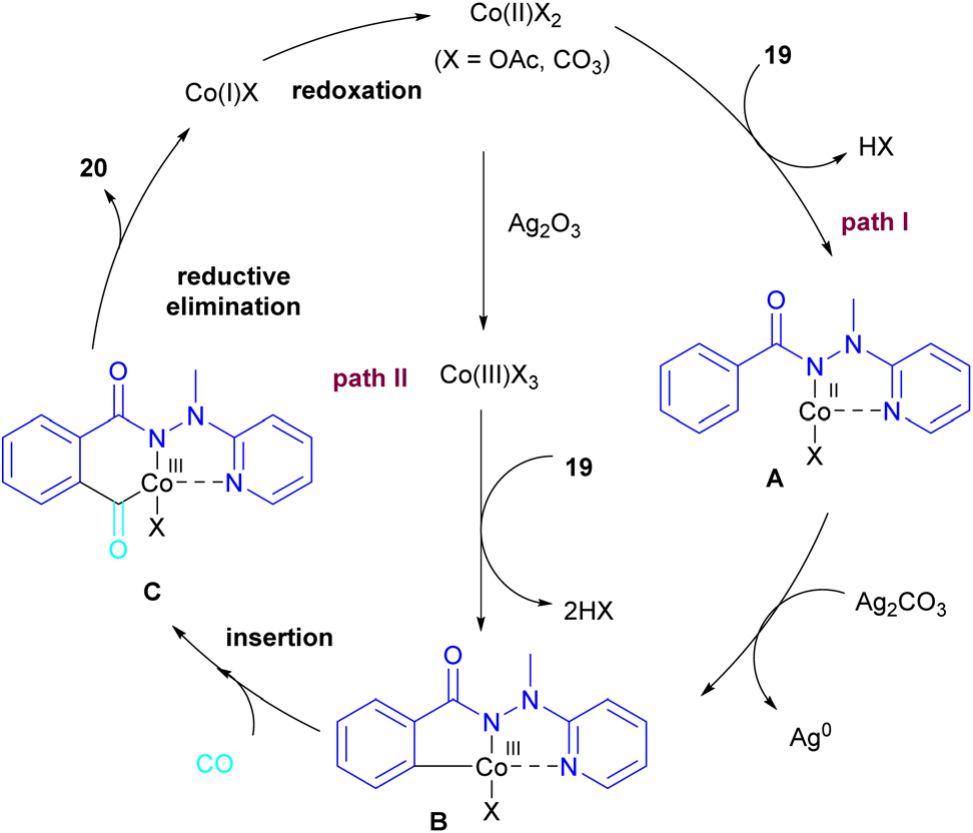
Catalytic cycle for Co-catalyzed carbonylation of benzoyl hydrazide derivatives.

In 2018, a cobalt-catalyzed synthesis of *N*-pyridyl phthalimide derivatives 20*via* the carbonylation of benzoyl hydrazides was described by the Zhai group (Scheme 10).^36^ By studying the deuterium labeling experiment of substrate 19 and the H/D exchange (CD_3_OD/CH_3_OH) experiment, the authors indicated that the C–H bond cleavage is not the rate-determining step, and this step is reversible. The removal of the pyridyl moiety could be easily carried out in the presence of RANEY® and H_2_. According to the mechanism, two pathways were proposed for the formation of cobaltacycle B. In path I, the coordination of hydrazide 19 with Co(ii), followed by the C–H bond activation led to the Co(iii) complex B in the presence of Ag_2_CO_3_. While in path II, first, the oxidation of Co(ii) to Co(iii) was promoted by Ag_2_CO_3_, which then underwent C–H activation with 19. Afterward, B underwent the insertion of CO to obtain the 6-membered cobaltacycle C, followed by reductive elimination to yield phthalimide 20 with the regeneration of the Co(i) species. The oxidation of Co(i) to the active Co(ii) species was carried out by Ag_2_CO_3_ (Scheme 11).

#### 2.1.4. Pd-catalyzed synthesis of phthalimides

**Scheme 12:**
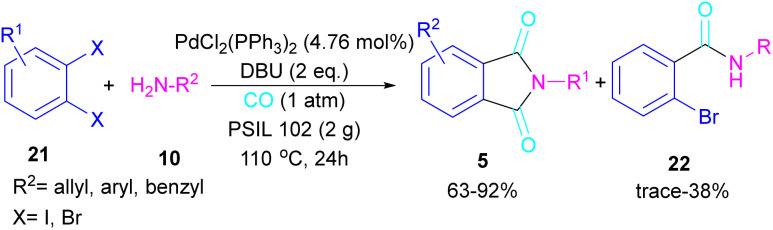
Pd-catalyzed double carbonylation of *o*-dihaloarenes with amines.

In 2005, Alterman and his team were able to synthesize phthalides and phthalimide through palladium-catalyzed carbonylation of aryl bromides using Mo(CO)_6_ as a CO source.^37^ The reaction mechanism was not reported in this work and the scope of phthalimide was limited to one derivative. In 2010, Alper and Cao developed a novel three-component reaction, involving *ortho*-dihaloarene, 21 amine 10 and gaseous carbon monoxide to construct *N*-substituted phthalimide derivatives 5 (Scheme 12).^38^ A palladium complex was used as a catalyst and the reaction was carried out in ionic liquid as a green solvent. To enhance the product yield of phthalimide rather than 2-halo benzamide byproduct, 1 atmosphere of CO should be used. A similar dicarbonylation reaction for the assembly of phthalimide scaffolds was reported in the same year.^39^ In this work, aryl/alkyl/heteroaryl amines reacted smoothly with *ortho*-dihalobenzene, *ortho*-iodobenzoinc acid and *ortho*-iodobenzoate under 90 psi of CO gas.

**Scheme 13:**
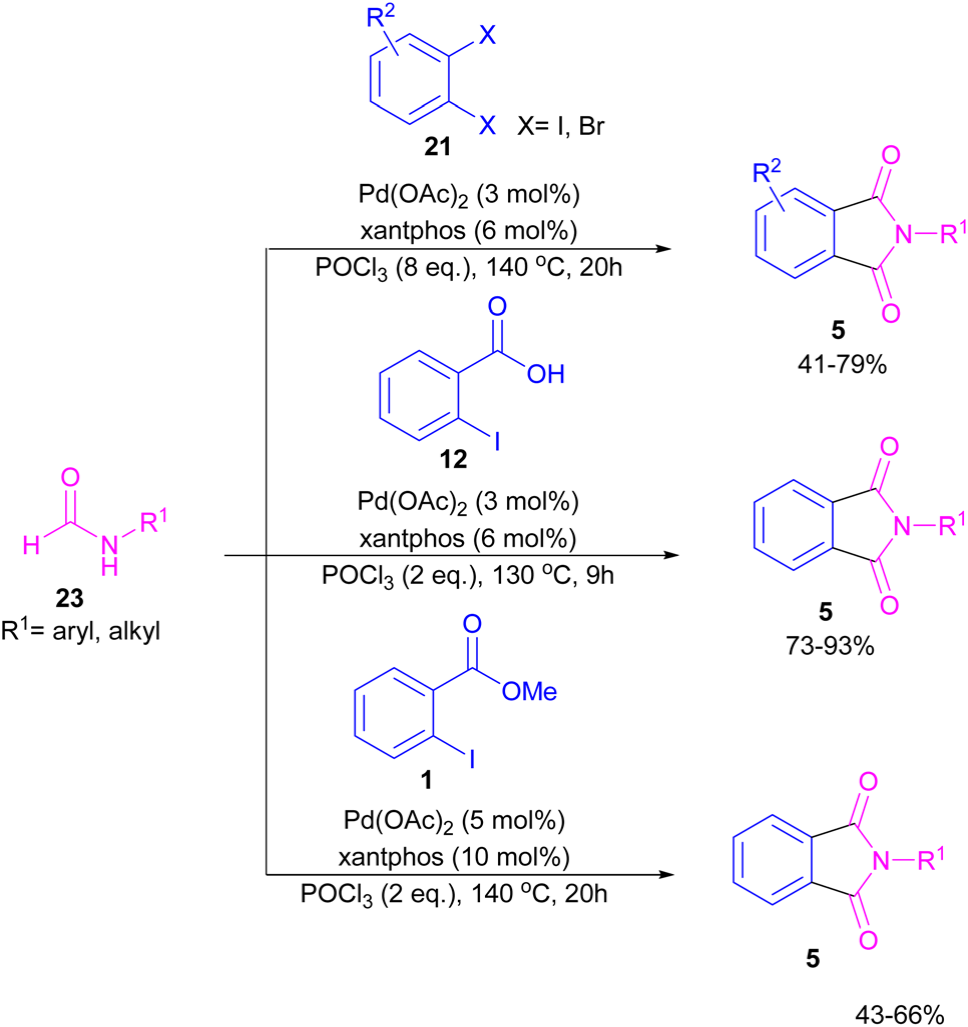
Pd-catalyzed cycloaminocarbonylation of *o*-haloarenes using formamides.

**Scheme 14:**
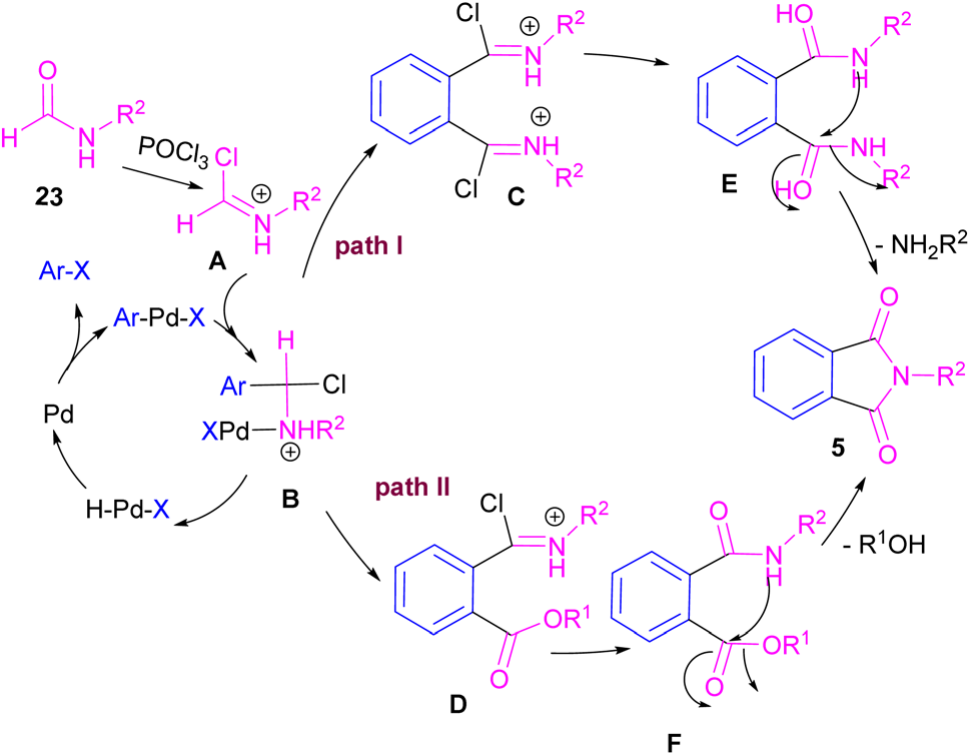
Possible mechanism for Pd-catalyzed cycloaminocarbonylation of *o*-haloarenes using formamides.

Interestingly, in the same year, a CO-free strategy was proposed for the synthesis of *N*-substituted phthalimides 5 from amides 23, and *ortho*-dihaloaryles 21, *ortho*-iodobenzoinc acid 12 or *ortho*-iodobenzoate 1 (Scheme 13).^40^ In this regard, Bhanage and co-workers applied palladium acetate as a catalyst and POCl_3_ as a Lewis acid in this double carbonylation. The authors proposed a possible reaction mechanism, which was initiated by the oxidative insertion of Pd into the C–X bond to obtain the aryl palladium intermediate 1, followed by the attack on the imminium salt A to form B. For *o*-dihaloarenes, this process was carried out for both the *ortho*-halo groups. Afterward, β-hydride elimination in B afforded intermediate C for the case of *ortho*-dihaloarenes, while for *ortho*-halo acid and *ortho*-halobenzoate D was the obtained intermediate. In the next stage, C led to E through the attack of the amide lone pair on the carbonyl group of the other *o*-amide, followed by intramolecular cyclization to form product 5. This product was furnished from F*via* the attack of lone pair the amide on acid or ester carbonyl group and subsequent intramolecular cyclization (Scheme 14).

**Scheme 15:**
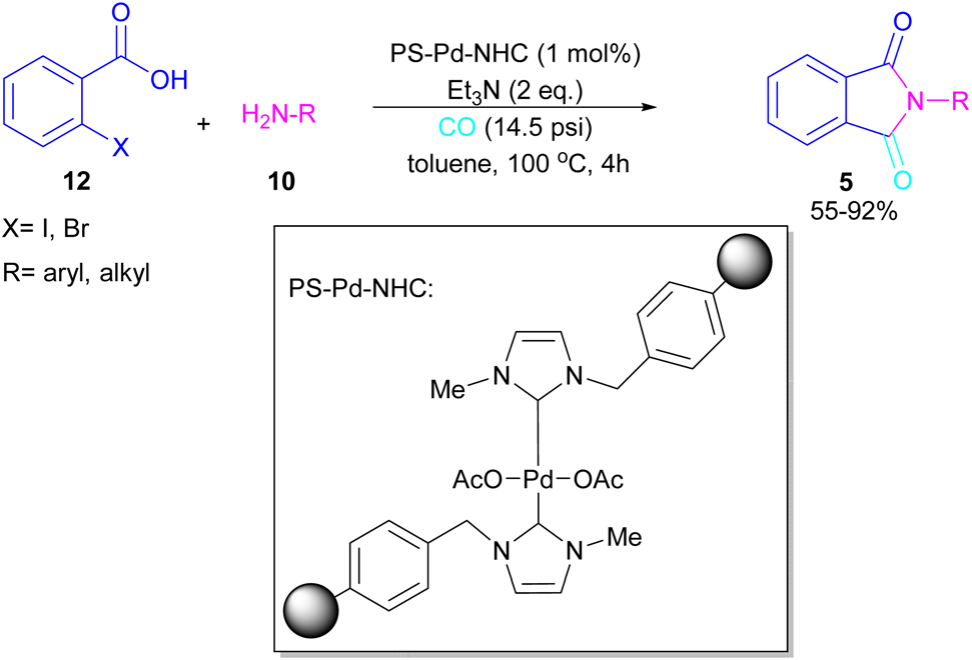
Cabonylative cyclization of *o*-halobenzoic acids for synthesis of *N*-phthalimides.

A polymer-supported palladium-N-heterocyclic carbine was introduced for the synthesis of *N*-substituted phthalimides 5 from *ortho*-halobenzoic acid 12, aryl/alkyl amines 10 and CO (Scheme 15).^41^ In this method, Bhanage and co-workers did not use POCl_3_ in the reaction and the decarboxylative cyclization of *ortho*-halobenzoic acid was carried out in the presence of a heterogeneous and reusable catalyst, which could be recovered for several cycles. In addition to *ortho*-halobenzoic acids, methyl *ortho*-iodobenzoate also gave the desired product in good yields (70–80%). In this synthetic method, 14.5 psi of CO was required.

**Scheme 16:**
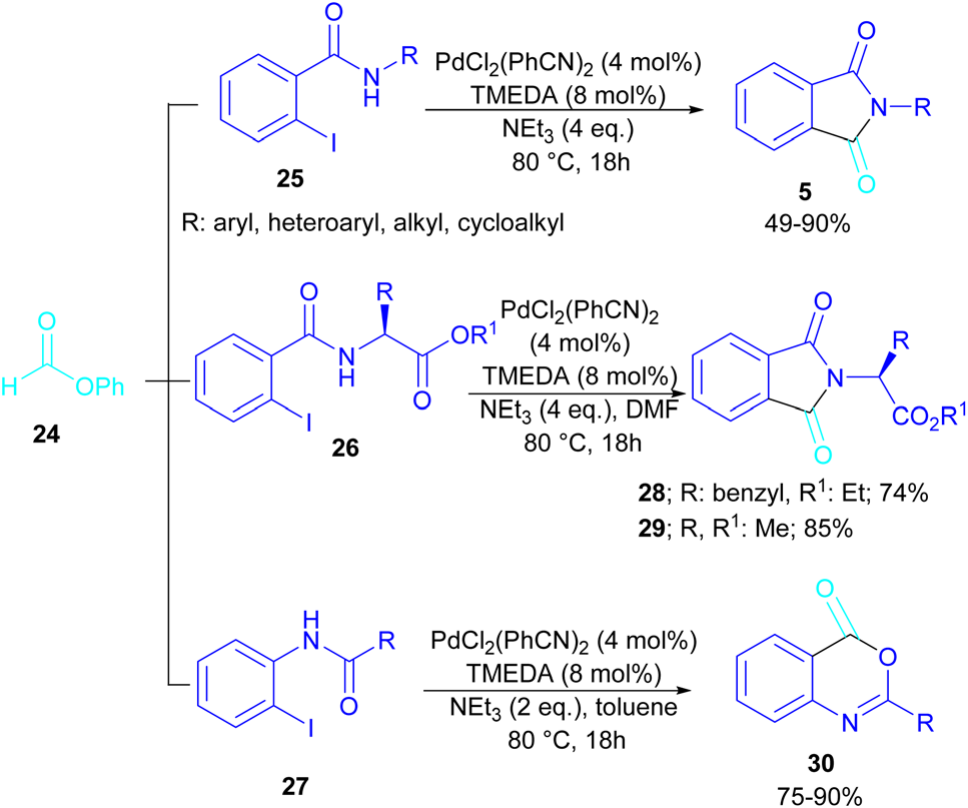
Pd-catalyzed carbonylative cyclization of *N*-substituted 2-iodobenzamides and 2-iodoanilides.

**Scheme 17:**
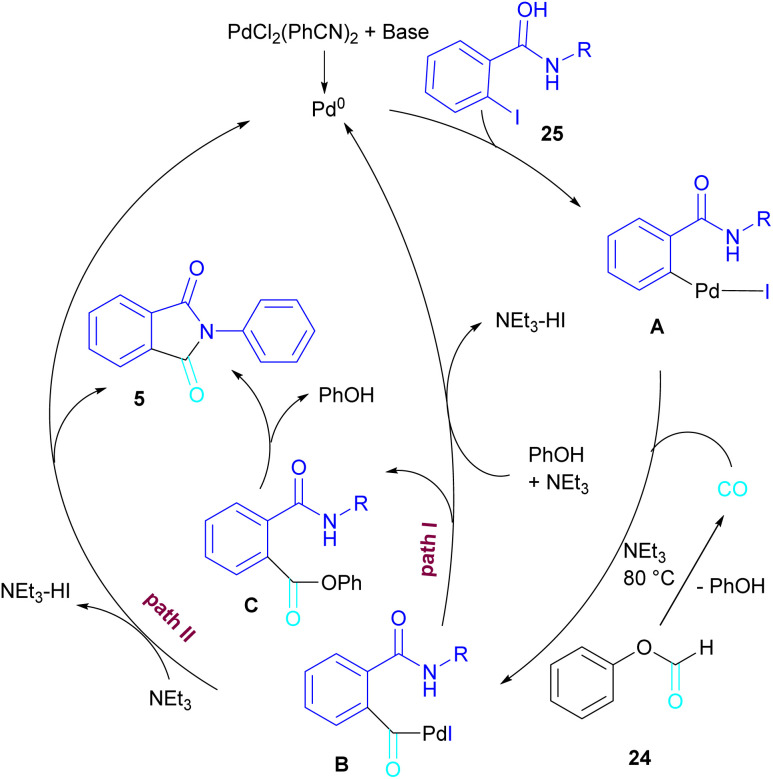
Possible mechanism for Pd-catalyzed carbonylative cyclization of *N*-substituted 2-iodobenzamides and 2-iodoanilides.

In 2015, Bhanage and his team synthesized a series of *N*-substituted phthalimides using a palladium catalyst (Scheme 16).^42^ In this context, they treated *N*-substituted 2-iodobenzamides 25 with phenyl formate in the presence of a palladium catalyst to obtain phthalimides 5 under solvent-free conditions. Whereas, the reaction of benzamides 26 with phenyl formate 24 needed solvent to proceed. The use of phenyl formate made this method unnecessary for the CO gaseous. A catalytic cycle was proposed for this transformation, which involved the oxidative addition of Pd(0) to 2-iodobenzamide 25 to obtain the arylpalladium intermediate A. In the meantime, phenyl formate was decomposed into CO under heat, which then reacted with A to form the acylpalladium intermediate B. In this stage, two possible pathways were considered for the production of phthalimide 5 from B. In path I, a phenoxycarbonylation of B, followed by an intramolecular annulation gave 5. While, a nucleophilic intramolecular attack occurred in B to form 5. Furthermore, the researchers showed that the use of this palladium catalytic system for the reaction of 2-iodoanilide 7 with phenyl formate 24 led to the benzoxazinone synthesis (Scheme 17). In the same year, a palladium-catalyzed synthesis of phthalimides was reported from 2-OTS benzamides and CO as a carbonyl source.^43^ Another palladium catalysis synthetic method for the assembly of phthalimides was reported by Sekar and co-workers.^44^ In this method, 2-iodobenzamides were treated with CO gaseous under a binaphthyl-supported palladium (Pd-BNP) catalyst as a heterogeneous and reusable catalyst. Also, the reaction of 2-bromobenzamide with oxirane as a CO source can lead to phthalimide in the presence of Pd/C.^45^

**Scheme 18:**
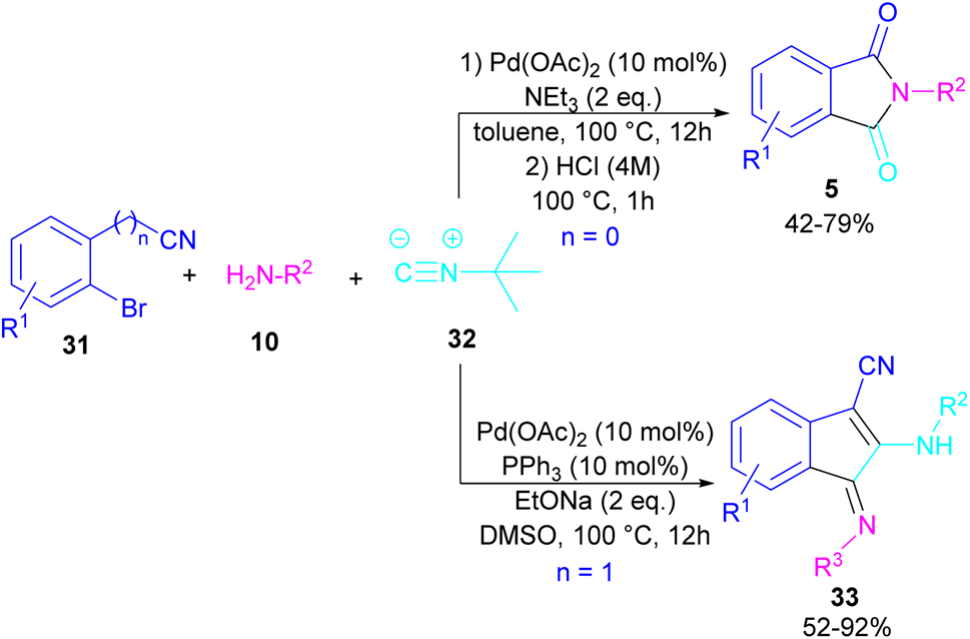
Pd-catalyzed synthesis of phthalimides and 1*H*-indenes *via* isocyanide insertion.

**Scheme 19:**
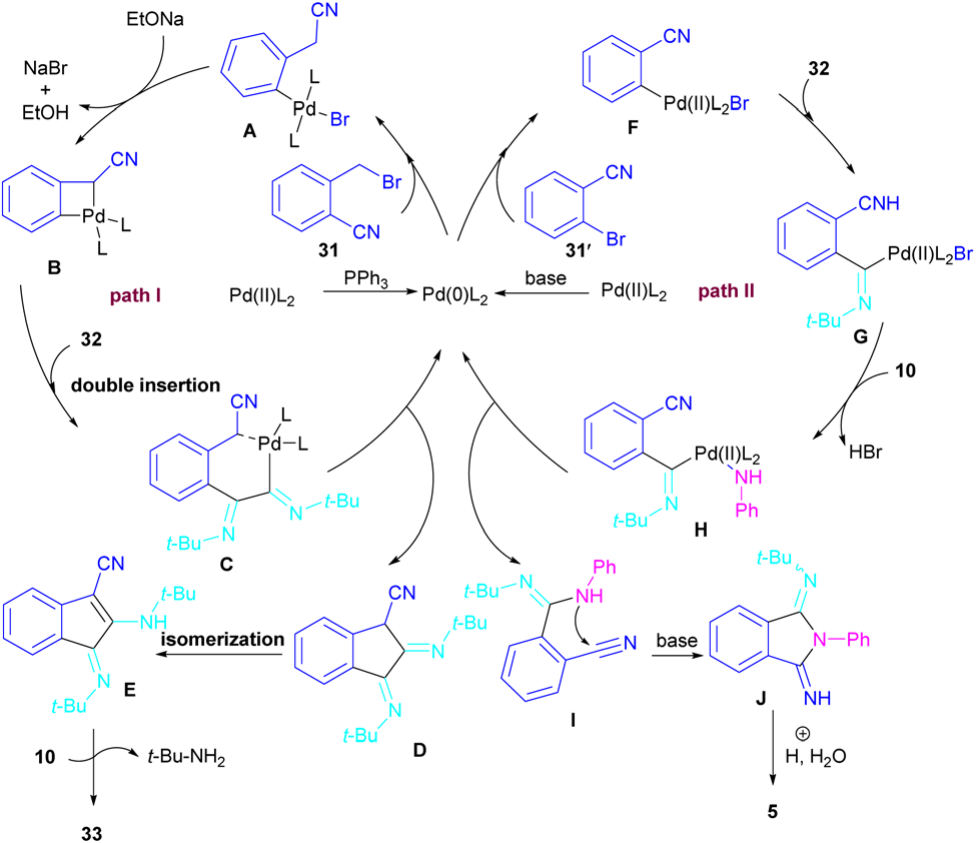
Possible mechanism for Pd-catalyzed synthesis of phthalimides and 1*H*-indenes *via* isocyanide insertion.

In 2017, a three-component reaction was extended for the preparation of phthalimides under palladium catalysis (Scheme 18).^46^ In this regard, bromobenzonitrile 31, isonitrile 32, and aromatic amines 10 were treated in the presence of Pd(OAc)_2_ and Et_3_N to produce a series of *N*-substituted phthalimides 5. By changing the bromobenzonitrile substrate (when *n* = 0) to 2-(2-bromophenyl)acetonitrile (when *n* = 1) and the replacement of NEt_3_ with PPh_3_, this multi-component reaction led to 1*H*-indenes as the final products. Two catalytic cycles were proposed for these transformations (Scheme 19). In the path I, oxidative addition of 31 to Pd(0) produced the aryl palladium species A, which was subjected to cyclopalladation to form the four-membered palladium cycle B. The subsequent double insertion of isocyanide 32 into the Pd–C bond resulted in intermediate C, which underwent reductive elimination to render intermediate D, followed by the isomerization towards intermediate E. Finally, an amine exchange between 10 and E delivered product 33. In path II, the oxidative addition of 31′ with L_2_Pd(0) led to F, followed by the insertion of 32 to render G. This intermediate then reacted with 10 to form intermediate H. Subsequent reductive elimination of H, followed by the nucleophilic attack of amidine to the nitrile generated intermediate J, which hydrolyzed to product 5 under acidic conditions.

**Scheme 20:**
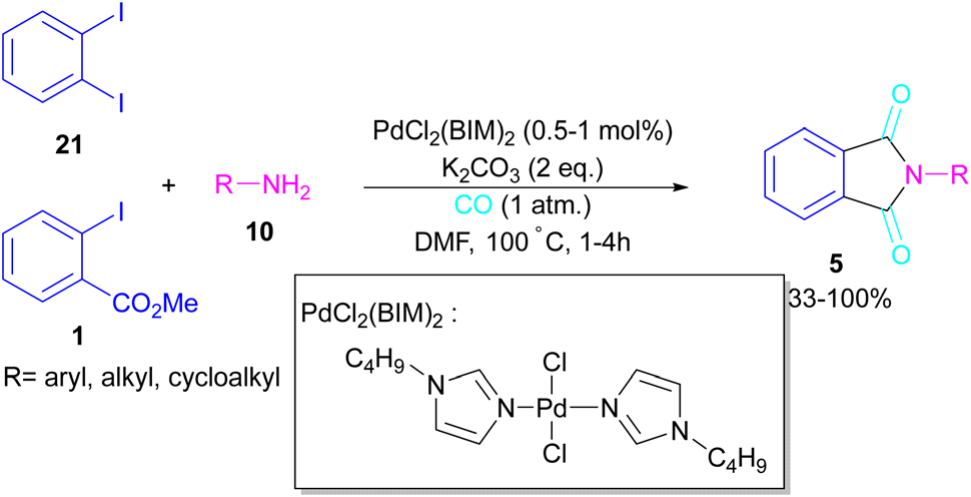
Pd-catalyzed reaction of 1,2-diiodoarenes with primary and secondary amines.

**Scheme 21:**
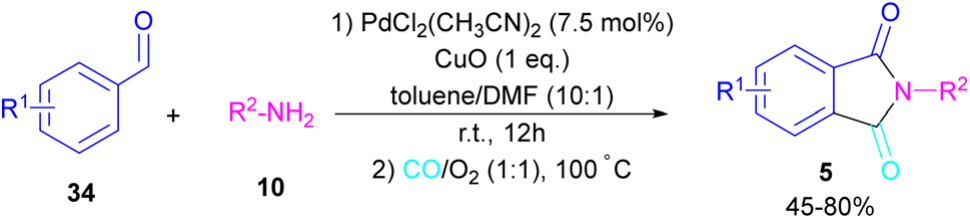
Pd-catalyzed aminocarbonylation of aldehydes.

**Scheme 22:**
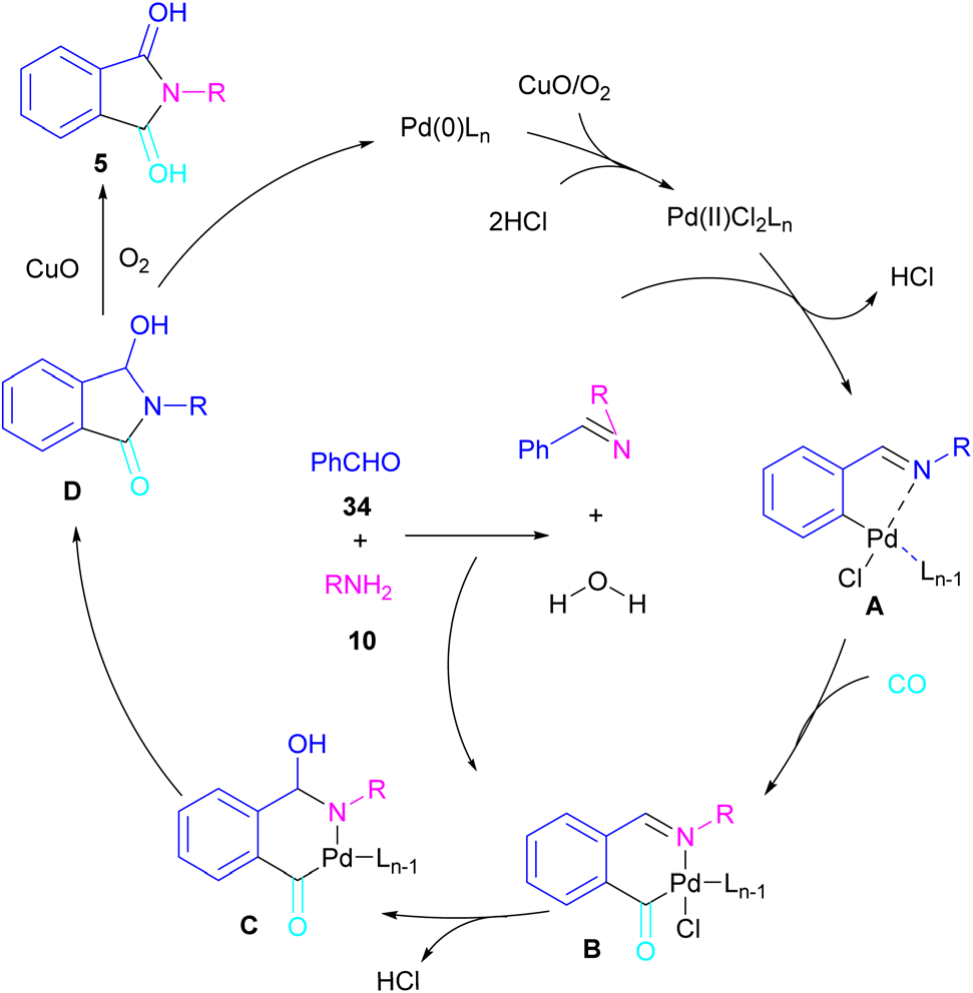
Possible mechanism for Pd-catalyzed aminocarbonylation of aldehydes.

In 2018, a new palladium catalyst was applied for the synthesis of phthalimide frameworks 5 (Scheme 20).^47^ The palladium catalyst with an imidazole ligand can act as an efficient catalyst in the reaction of *ortho*-diiodobenzenes 21 or methyl 2-iodobenzoate 1 with amines under a CO atmosphere. The presence of imidazole ligand can promote the catalytic activity and the reaction efficiency. It is noteworthy that a low amount of this catalyst could catalyze the aminocarbonylation of 1,2-diiodoarenes at a shorter reaction time compared to other palladium catalysts (6–36 h), and also in lower pressure of CO than other related reports. Another palladium catalyst was used for the aminocarbonylation of aryl aldehydes 34 by amines 10 and CO gas (Scheme 21).^48^ In this method, the imine and H_2_O generated from the condensation of amine and aldehyde, acted as a directing group and a nucleophile, respectively. As shown in Scheme 22, the mechanism started with the imine-assisted C–H activation process to obtain intermediate A. Then, CO was inserted into the C–Pd bond to form intermediate B. In this step, H_2_O attacked the C=N bond to give intermediate C. Reductive elimination of C led to compound D, which was further oxidized in the presence of CuO and O_2_ to yield product 5. The generated Pd(0) could be reoxidized to the active Pd(ii) catalyst. In another report, palladium acetate was used in the reaction of 2-iodobenzamides and formic acid to form the phthalimide derivatives.^49^ Formic acid served as a carbonyl source in this reaction.

**Scheme 23:**
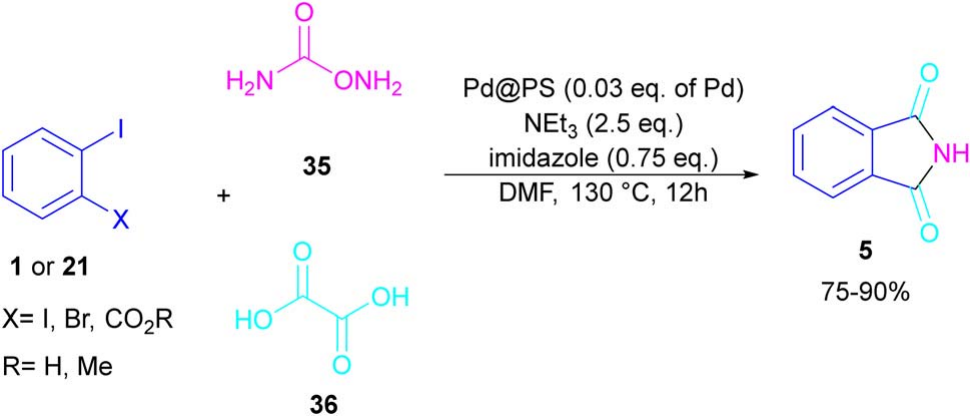
Pd-catalyzed reaction of aryl iodides with ammonium carbamate and oxalic acid.

**Scheme 24:**
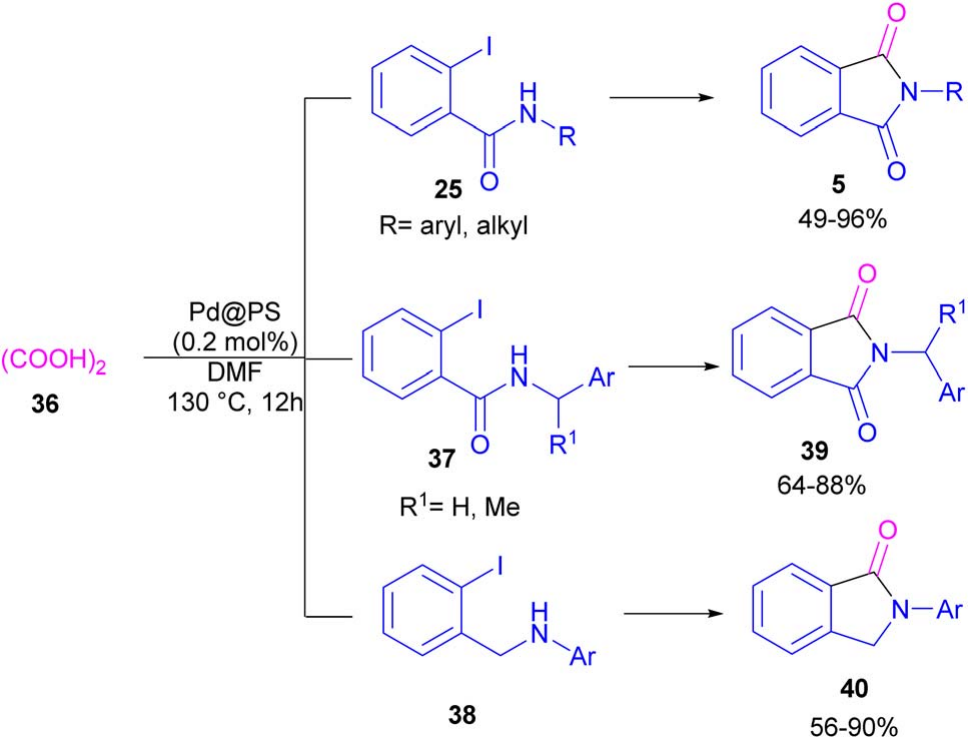
Pd@PS-catalyzed catalyzed reactions of 2-iodobenzamides and 2-iodobenzylanilines with oxalic acid.

**Scheme 25:**
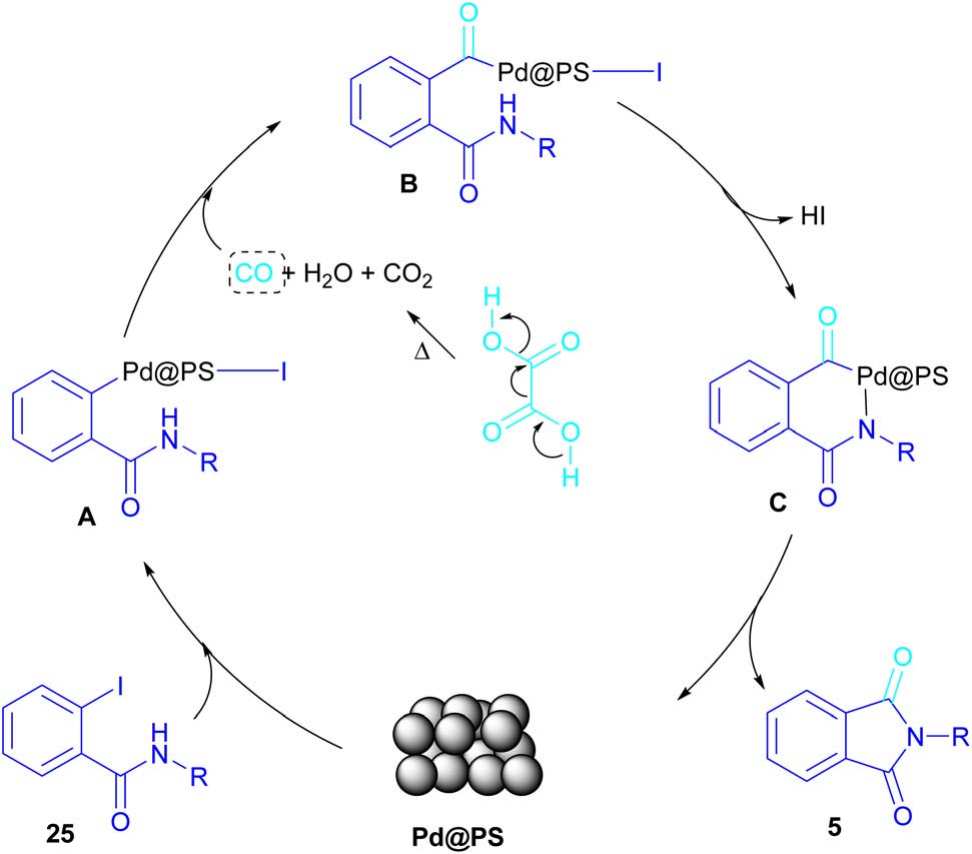
Possible mechanism for Pd@PS-catalyzed reactions of 2-iodobenzamides and 2-iodobenzylanilines with oxalic acid.

In 2020, Das *et al.* used a new polystyrene-supported palladium (Pd@PS) nanoparticle (NPs) catalyst for the assembly of phthalimides 5 (Scheme 23).^50^ In their method, 1,2-dihalobenzene 21, or 2-halobenzoates 1 reacted with ammonium carbamate 35 and oxalic acid 36. The protocol has the advantages of a heterogeneous catalyst, the use of oxalic acid instead of CO gaseous, and the use of ammonium carbamate as an amine synthon. In another work, this group treated 2-iodobenzamides and 2-iodobenzylanilines 25 with oxalic acid 36 to achieve a wide spectrum of phthalimides 5 and isoindolinones 40 (Scheme 24).^51^ polystyrene supported-palladium (Pd@PS) nanoparticles were used for this transformation, which could be recovered and reused for six cycles without significant loss of catalytic activity. Briefly, the oxidative addition of Pd@PS to the C–I bond of 25 generated the Pd complex A. Meantime, oxalic acid decomposed under thermal conditions into CO, which coordinated with A to form the acyl Pd complex B. Intramolecular nucleophilic attack of the N-atom on the Pd center afforded the cyclized intermediate C, followed by reductive elimination to yield product 5 and regenerate Pd@PS catalyst to restart the next cycle (Scheme 25).

**Scheme 26:**
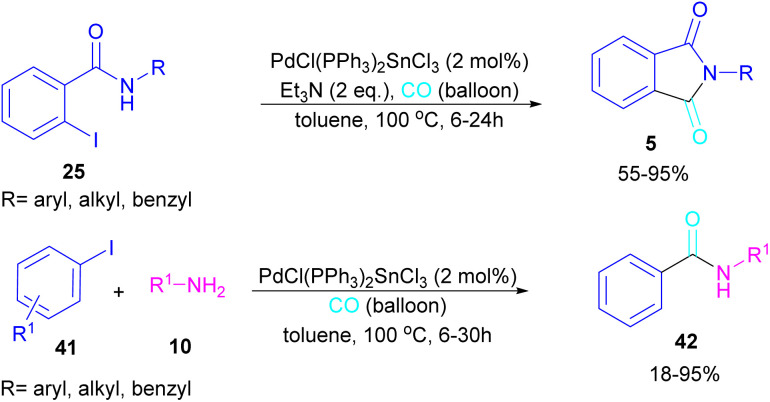
Pd-catalyzed aminocarbonylation reaction of aryl iodides with amines.

In 2024, another group reported the preparation of phthalimides 5 and amides 42 from 2-iodobenzamide 25 and iodobenzene 41, respectively catalyzed by PdCl(PPh_3_)_2_SnCl_3_ (Scheme 26).^52^ In this method, a CO balloon was used as the carbonyl source and the final products were obtained in moderate to high yields. It is noteworthy that the combination of Pd with Sn as a catalyst was necessary for the reaction to proceed. In addition, the synthesis of a COX inhibitor, such as *N*-(3,4,5-trimethoxyphenyl)phthalimide was also obtained in this work.

**Scheme 27:**
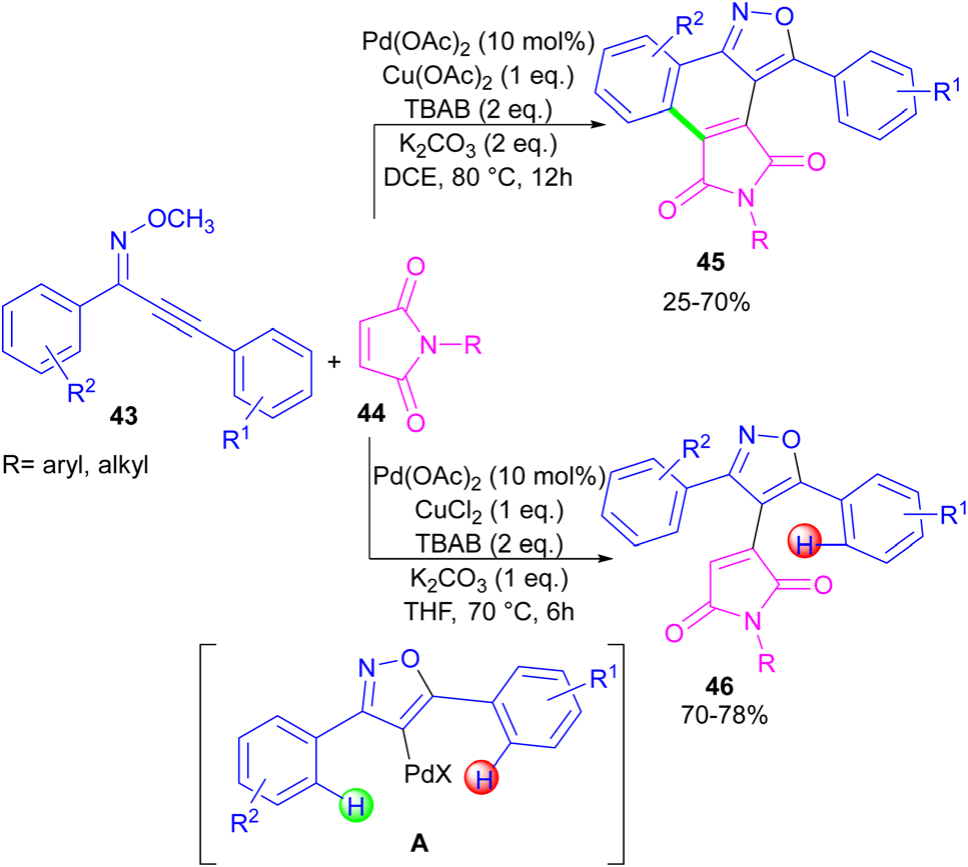
Pd-catalyzed reaction of oxime ethers and maleimides.

In the same year, a novel class of phthalimide scaffolds was synthesized through palladium-catalyzed double C–H activation/annulation of alkynyl-oxime ethers 43 using maleimide 44 (Scheme 27).^53^ By changing some parameters in the reaction, such as the copper oxidant and the solvent, two different kinds of products were obtained under palladium catalysis. Where 1.0 equivalent of Cu(OAc)_2_ and 2.0 equivalents of K_2_CO_3_ were used in DCE as a solvent, C–H activation of alkynyl-oxime ether by Pd(ii) and subsequent annulation resulted in the phthalimide product. This reaction proceeded through the formation of the alkenyl-palladium intermediate A by the 5-endo-dig cyclization of oxime ether 43 in the presence of Pd(ii), followed by regioselective 1,4-Pd migration to the C–H bond of another aryl ring. While 1.0 equivalent of CuCl_2_, and 1.0 equivalents of K_2_CO_3_ as well as THF as a solvent were needed for the synthesis of the maleimide product 46. In this case, after the C–H activation process leading to intermediate A, the coordination and insertion of maleimide 43 gave product 46.

#### 2.1.5. Rh-catalyzed synthesis of phthalimides

**Scheme 28:**
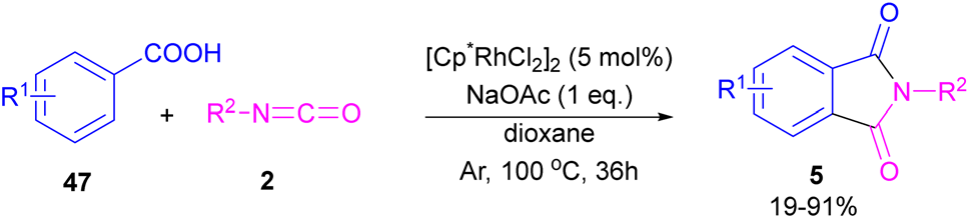
Rh-catalyzed synthesis of *N*-substituted phthalimides from isocyanates and benzoic acids.

**Scheme 29:**
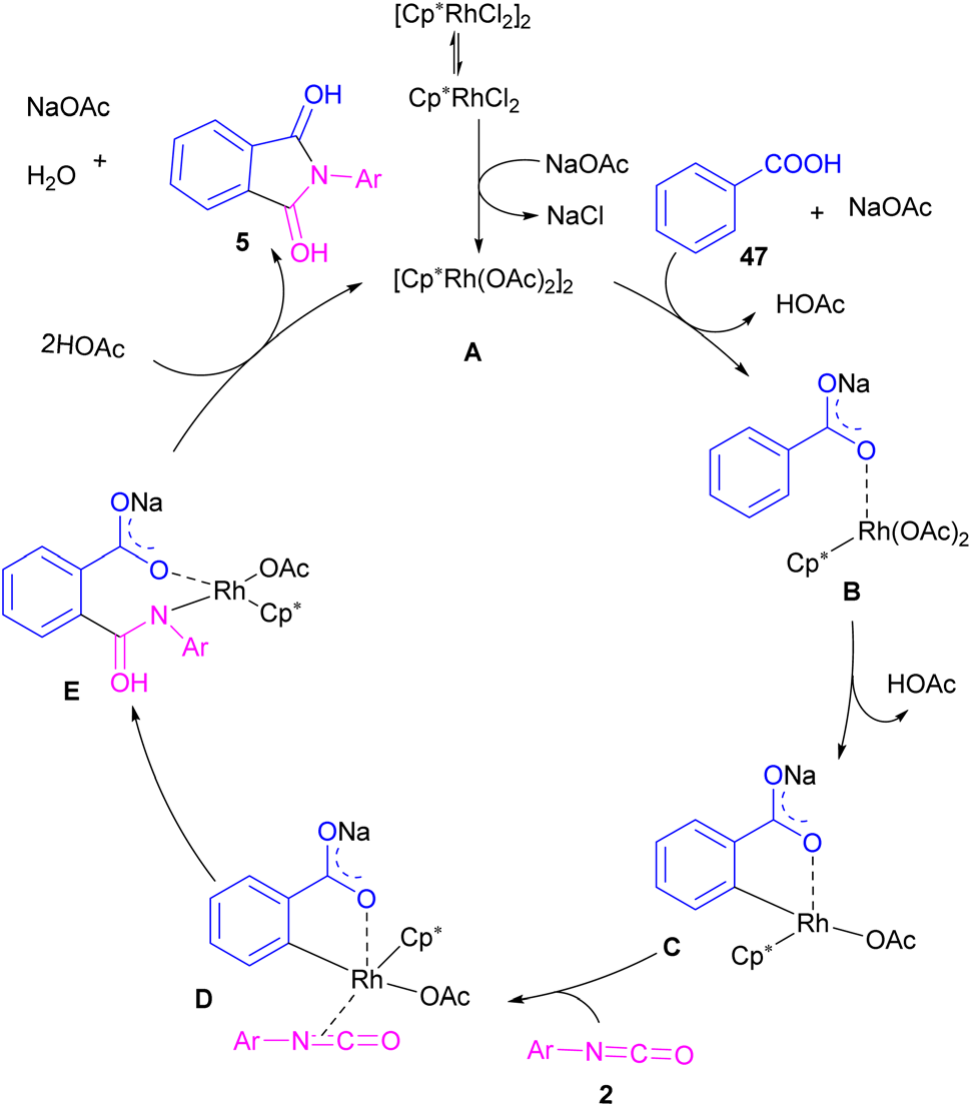
Catalytic cycle for Rh-catalyzed reaction of isocyanates and benzoic acids.

In 2014, the Li research group developed a rhodium catalysis system for the construction of phthalimides 5 from the cyclization reaction between benzoic acids 47 and isocyanate 2 (Scheme 28).^54^ The reaction involved *ortho*-C–H activation of benzoic acids and subsequent amination. Not only aryl isocyanates, but also alkyl isocyanates reacted smoothly to afford *N*-substituted phthalimides. According to the mechanism in Scheme 29, a ligand exchange was carried out between NaOAc and [Cp*RhCl_2_]_2_ to obtain the active species A, which was then coordinated to the carboxylic oxygen to render the rhodium benzoate B. Direct C–H bond activation in B afforded the rhodacycle C, which underwent the coordination with isocyanate 2, followed by the insertion into the Rh–C bond to form the rhodium alkoxide E. Intramolecular dehydration of E gave product 5 and regenerated the active catalyst A. By the kinetic isotope effect (KIE) experiment, the authors indicated that the C–H activation is the rate-determining step and proceed through the electrophilic aromatic substitution (S_E_Ar) mechanism.

**Scheme 30:**
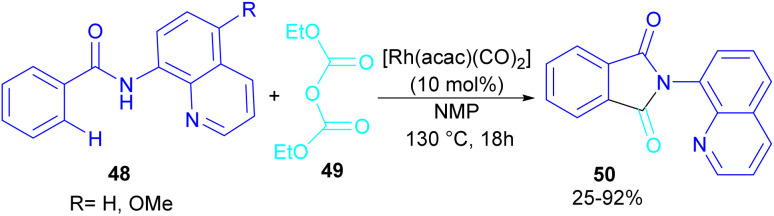
Rh-catalyzed carbonylation of benzamides with diethyl dicarbonate.

**Scheme 31:**
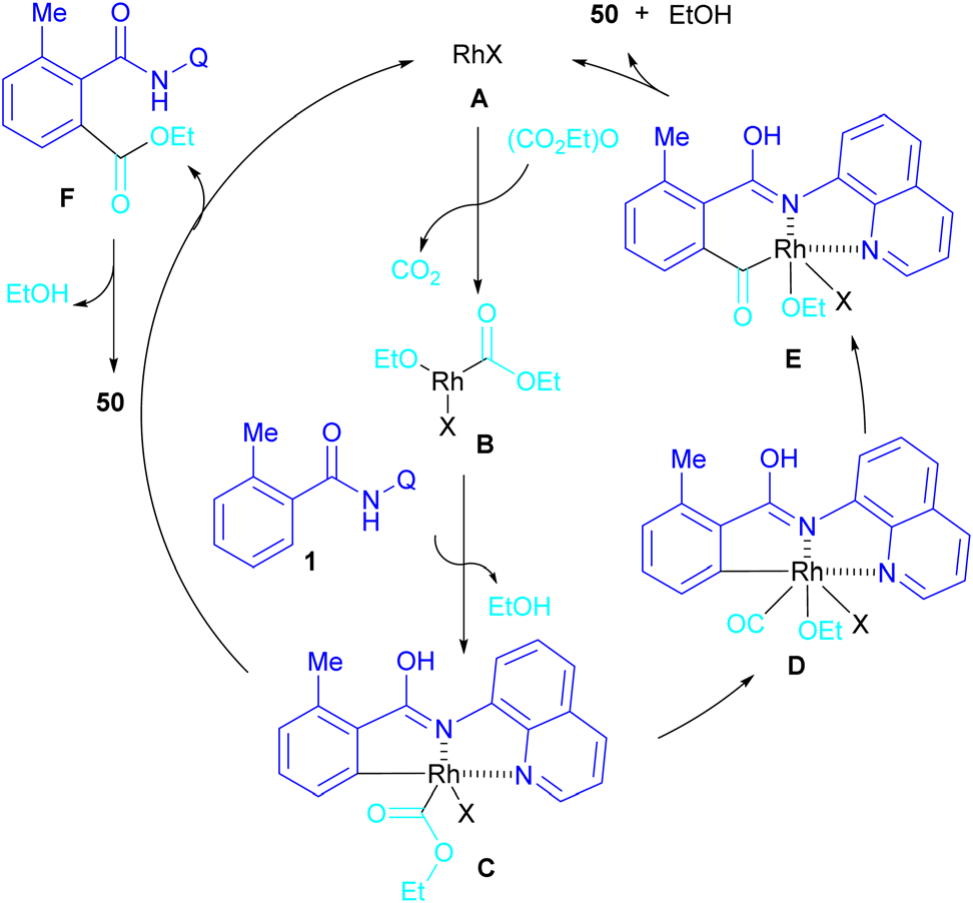
Catalytic cycle for Rh-catalyzed carbonylation of benzamides with diethyl dicarbonate.

In 2024, Suzuki *et al.* constructed a series of *N*-quinolyl phthalimides 50 through C–H activation/carbonylation of 8-aminoquinoline benzamides 48 with diethyl dicarbonate 49 (Scheme 30).^55^ The procedure was performed in the absence of CO and additive and diethyl dicarbonate served as a carbonyl synthon. As shown in Scheme 31, the oxidative addition of Rh to 49 gave rhodium ethoxide B. Then, C–H bond activation of 48 by B occurred to obtain the five-membered rhodacycle C. At this stage, two possible pathways were suggested by the authors. In path I, C underwent reductive elimination to obtain *ortho*-(ethoxycarbonyl) benzamide F with concomitant regeneration of the Rh catalyst. Afterward, product 50 was furnished through intramolecular *N*-nucleophilic attack. While, in path II, C underwent a CO de-insertion and subsequent C(aryl)–Rh insertion to yield acyl rhodium intermediate E, followed by reductive elimination to deliver product 50 and an EtOH molecule. The deuterium labelling experiment using deuterated ethanol revealed that the C–H activation step is reversible and not rate-limiting step due to the kinetic isotope effect experiment.

#### 2.1.6. Ru-catalyzed synthesis of phthalimides

**Scheme 32:**
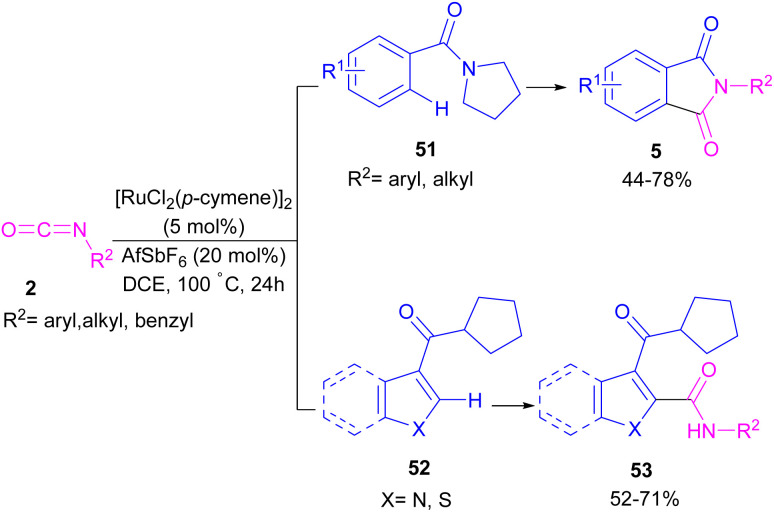
Ru-catalyzed C–H activation of benzamides with isocyanates.

**Scheme 33:**
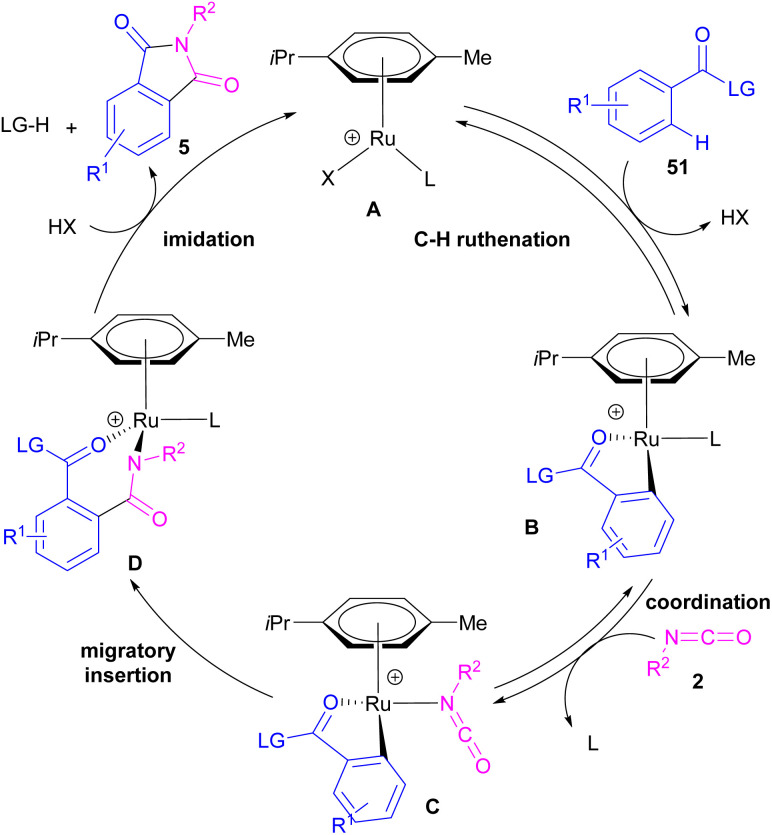
Ru-catalyzed C–H activation of benzamides with isocyanates.

Ackermann and De Sarkar in 2014 explored C–H activation of benzamides with isocyanates in the presence of a ruthenium(ii) complex (Scheme 32).^56^ In this Ru-catalyzed C–H activation, the reaction of substituted benzamides 51 with isocyanate 2 led to phthalimides 5, while the use of furan or pyrrole substrates 52 produced the uncyclized amides 53 as the final products. Mechanistic studies revealed a reversible C–H bond metalation of amide 51 by Ru(ii) complex, followed by the coordination of isocyanate 2 to form intermediate C. A migratory insertion in C gave intermediate D. Afterward, D underwent a proto-demethylation process to render diamide 5, or direct imidation to deliver phthalimide 5 along with the regeneration of the cationic ruthenium species A (Scheme 33).

### 2.2. Metal-free synthesis of phthalimides

Metal-free synthesis of phthalimide derivatives can be carried out under organocatalysis, base catalysis, or acid catalysis methods.^57–61^ Compared to transition metal catalysts, these methods offer a greener appeal. For example, organocatalysis approaches are readily available, cost-effective, low toxic, environmentally friendly and insensitive to moisture or oxygen, which makes them a suitable and promising route for preparing pharmaceuticals over metal catalysts.^62–65^

**Scheme 34:**
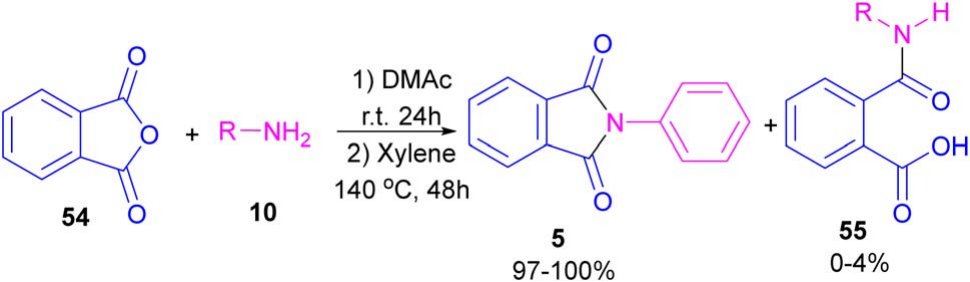
The amidation of phthalic anhydride using amines.

**Scheme 35:**
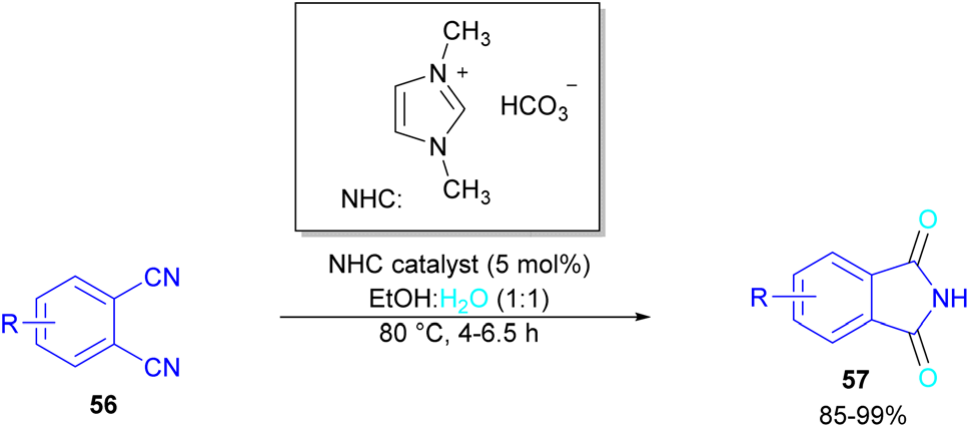
NHC-catalyzed-synthesis of phthalimides from 1,2-benzenedinitriles.

**Scheme 36:**
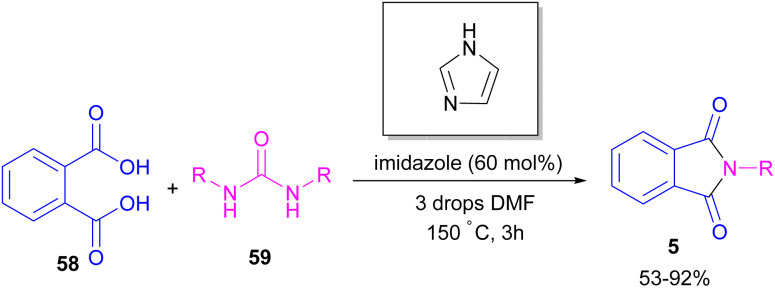
Reaction of *N*,*N*′-disubstituted urease, and phthalic acid catalyzed by imidazole.

In 2004, Li and co-workers reported the synthesis of phthalimide 5 from the amidation of phthalic anhydride 54 by amine 10 under metal-free conditions (Scheme 34).^66^ The products were obtained in excellent yields with a trace amount of the uncyclized byproduct 55. In 2013, an imidazole catalyst was utilized for the synthesis of phthalimides 57 from 1,2-benzenedinitriles 54 (Scheme 35).^67^ The reaction was carried out through double hydration of 1,2-benzenedinitriles, followed by intramolecular cyclization. Imidazole can catalyze the reaction of *N*,*N*′-dialkyl- or *N*,*N*′-diaryl-urease series 59 with phthalic acid 58 towards *N*-substituted phthalimides 5 (Scheme 36).^68^ Imidazole can activate the carbonyl moiety in phthalic acid for further attack of urea. Phthalimides were obtained in moderate to high chemical yields.

**Scheme 37:**
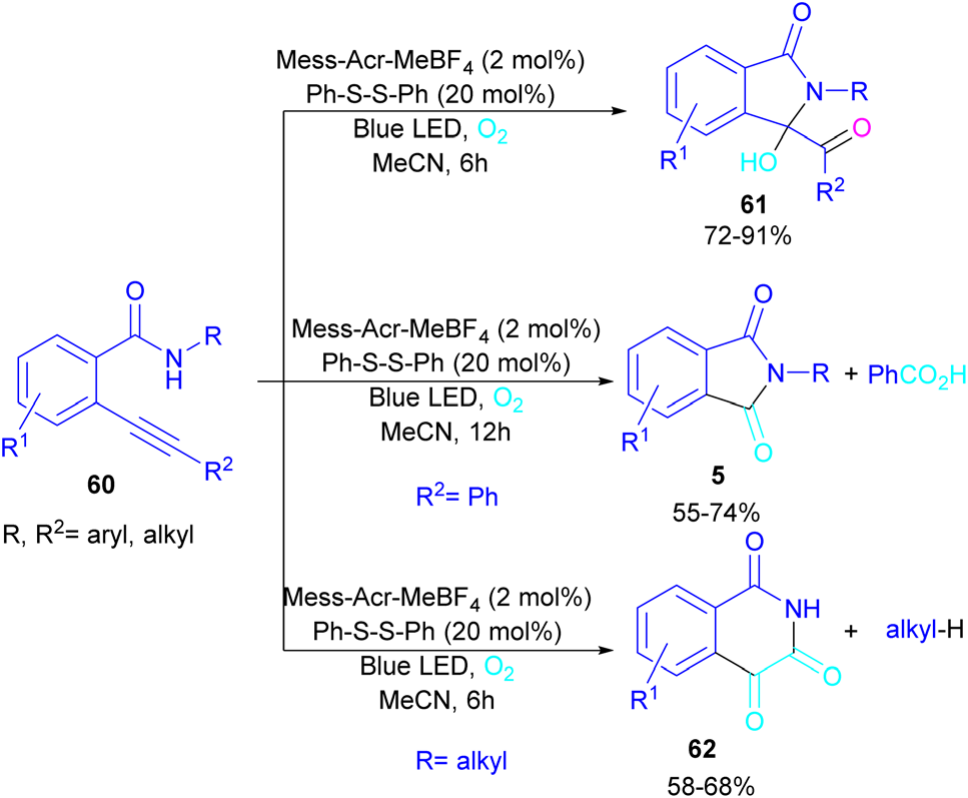
Visible light-promoted synthesis of isoquinoline-1,3,4(2*H*)-triones, 3-hydroxyisoindolin-1-ones, and phthalimides.

**Scheme 38:**
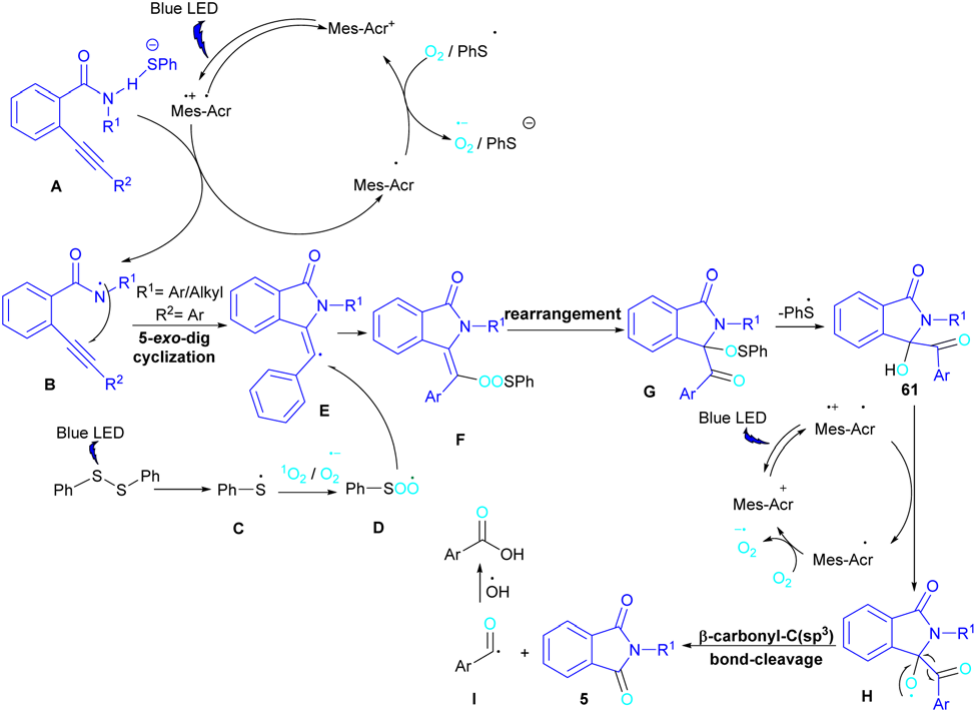
Tentative mechanism for visible light-promoted synthesis of isoquinoline-1,3,4(2*H*)-triones, 3-hydroxyisoindolin-1-ones, and phthalimides.

In 2022, Anandhan and co-workers were able to control the radical cyclization cascade for the preparation of 3-hydroxyisoindolin-1-ones 61, phthalimides 5 and isoquinoline-1,3,4(2*H*)-triones 62 (Scheme 37).^69^ Two possible routes were proposed for the cyclization of *ortho*-alkynylated benzamides under visible light irradiation. 5-Exo-dig cyclization led to 3-hydroxyisoindolin-1-ones 61 after 6 hours while increasing the reaction time to 12 hours resulted in the elimination of benzoic acid to obtain phthalimide derivatives 5. On the other hand, isoquinoline-1,3,4(2*H*)-triones 62 were constructed *via* 6-endo-dig cyclization. According to the mechanism of the synthesis of phthalimides, Acr+-Mes was excited to Acr˙-Mes˙+ under visible light, followed by a SET with *ortho*-alkynylbenzamide A access to the amidyl *N*-radical B and Acr˙-Mes. The Acr˙-Mes reduced O_2_ or the PhS radical C to form photocatalyst Acr+-Mes. When R^2^ = aryl alkyne, 5-exo-dig cyclization of radical B with alkynes led to a cyclized vinyl radical E. Meantime, thiylperoxyl radical D was produced by homolytic cleavage of PhSSPh under visible light irradiation, followed by addition with 1O_2_ or O_2_˙−, and then the addition with vinyl radical E to generate the cyclic intermediate F. Next, the homolytic O–O bond cleavage in F gave G, which underwent a radical transfer, and the elimination of the thiophenyl radical to deliver product 61. In the next stage, compound 61 was converted to the intermediate β-carbonyl alkoxyl radical H in the presence of the Acr+-Mes photocatalytic cycle. This intermediate was then subjected to the β-carbonyl-C(sp^3^) bond cleavage to form product 5 and the acyl radical I. On the other hand, I could be quenched by ˙OH to obtain acid 5′ (Scheme 38).

**Scheme 39:**
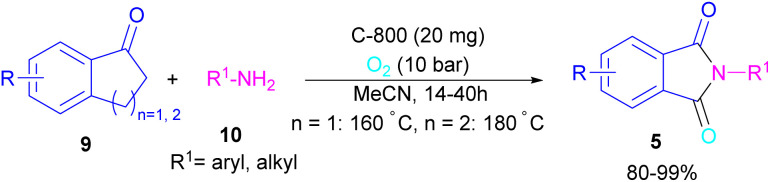
Carbon-catalyzed amidation of cyclic ketones towards phthalimides.

**Scheme 40:**
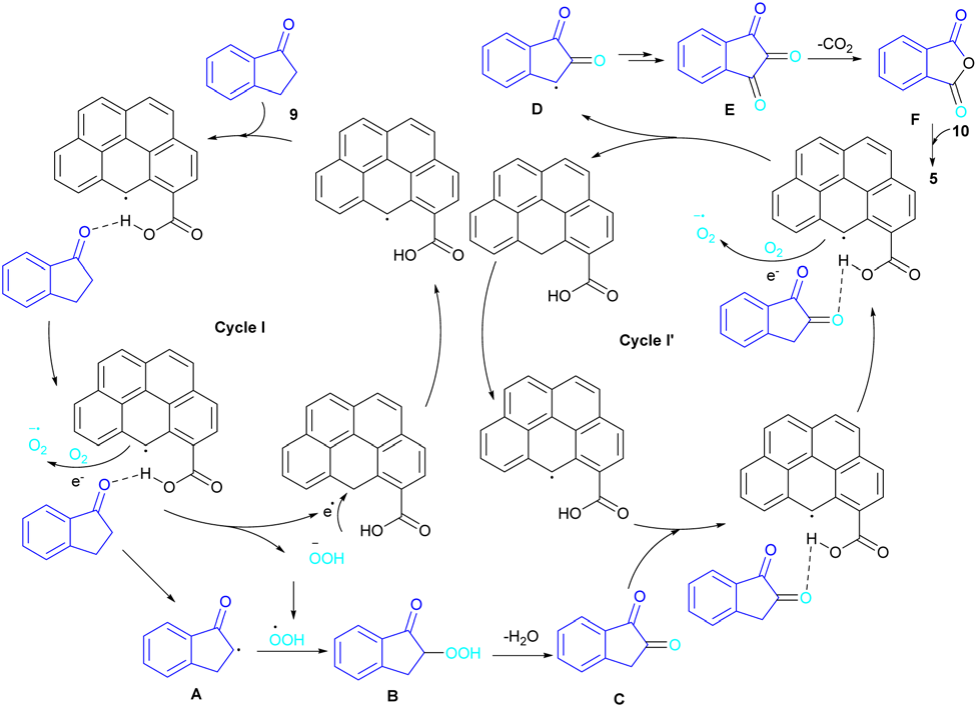
Catalytic cycles for carbon-catalyzed amidation of cyclic ketones towards phthalimides.

In 2022, a new carbon catalyst for the synthesis of phthalimides 5 was proposed by Zhang *et al.* (Scheme 39).^70^ For this purpose, first, they prepared a C-800 catalyst with a microporous surface area and a microporous volume and then used it in the reaction of cyclic ketones 9 and amines 10 under the O_2_ atmosphere. The mechanism was started with the initial absorption of O_2_ on the defective carbon and 1-indanone 9 on the COOH group of the catalyst. The electron transfer from the free electrons at the edge carbon to O_2_ led to ˙O_2_^−^ species, which could be captured ˙H from α-H in 1-indanone 9 to produce HOO^−^ species and regenerate one electron back to the catalyst to restart the next cycle with the formation of ˙OOH, simultaneously. The combination of ˙OOH with the carbon radical A gave intermediate B, which released one H_2_O molecule to form 1,2-indandione C. In this step, β-CH_2_ was oxidized into the C–O group to give 1,2,3-indantrione E*via* the same pathway. The extrusion of CO_2_ from 1,2,3-indantrione produced phthalic anhydride F, followed by an amidation process to deliver phthalimide 5 (Scheme 40). No need for metal, and CO as the carbonyl source and the use of O_2_ as the sole oxygen source makes this method sustainable and eco-friendly.

**Scheme 41:**
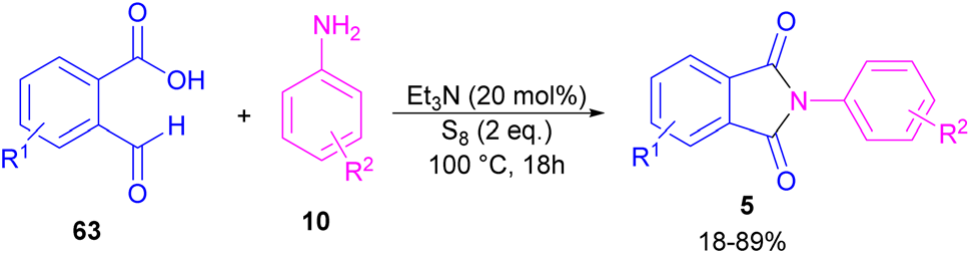
Reaction of 2-formylbenzoic acid and aniline.

**Scheme 42:**
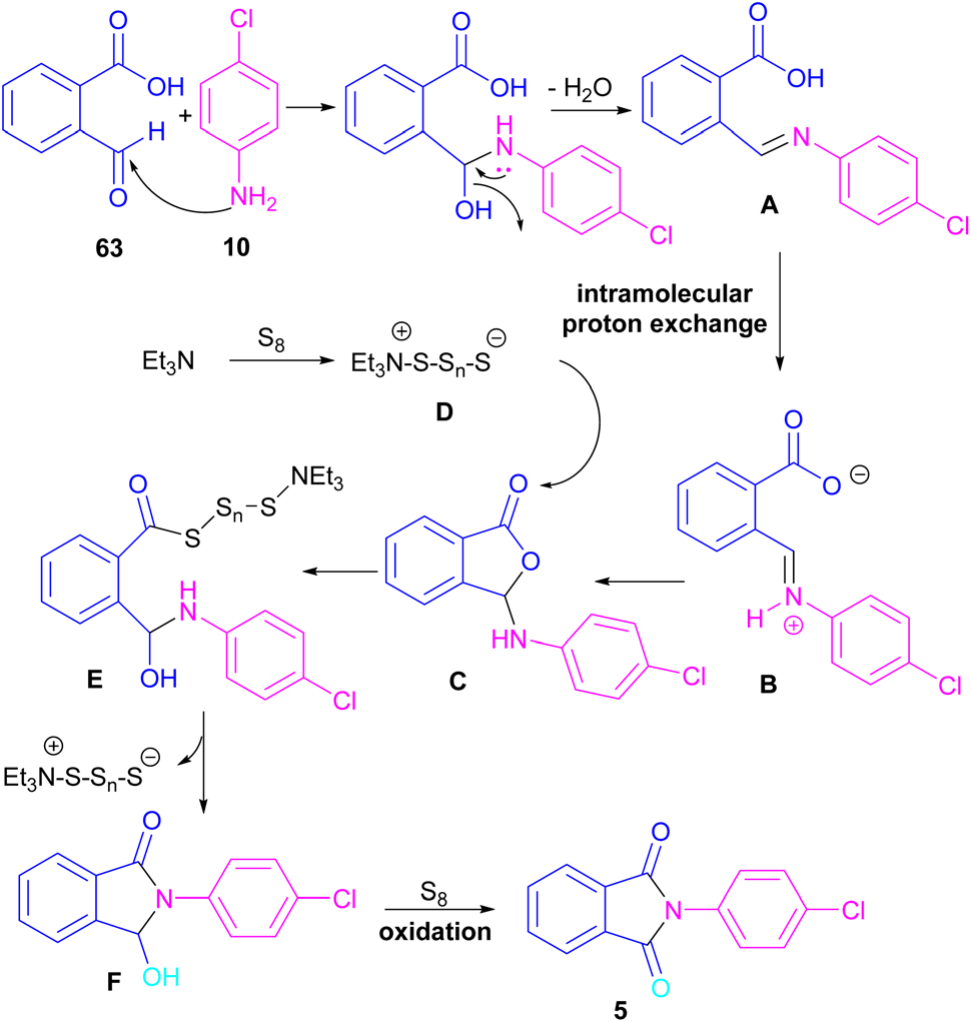
Possible mechanism for reaction of 2-formylbenzoic acid and aniline.

Another metal-free synthesis of *N*-aryl phthalimides was reported by the Mahdavi research team in 2022 (Scheme 41).^71^ A series of 2-formylbenzoic acids 63 and aryl/heteroaryl amines 10 reacted in the presence of Et_3_N as a base and S_8_ as an oxidant. In addition, *N*-benzyl phthalimides and N–H phthalimides were also obtained in 18% and 44%, respectively. In general, the reaction involved the initial condensation of 2-formylbenzoic acid 63 and amine 10 to form imide A, followed by an H-shift to obtain intermediate B. Through an intramolecular nucleophilic *O*-attack, intermediate C was formed, which was attacked by sulfur anion D to yield thioester E. Next, E underwent an intramolecular nucleophilic attack of nitrogen on the carbonyl to generate intermediate F, followed by further oxidation by S_8_ to deliver product 5 (Scheme 42).

**Scheme 43:**
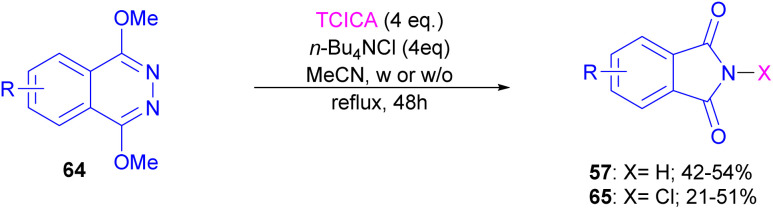
Synthesis of phthalimides from 1,4-dimethoxyphthalazines.

**Scheme 44:**
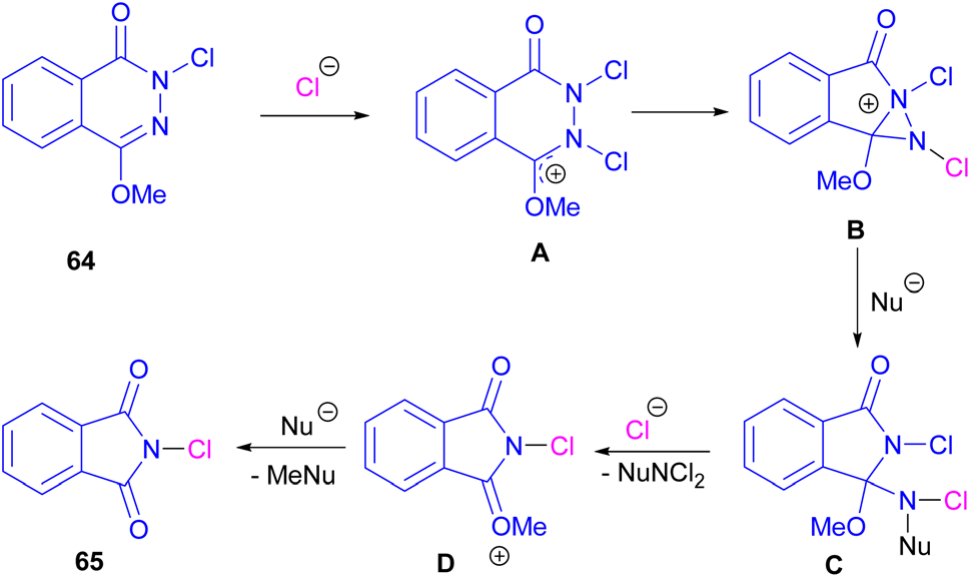
Plausible mechanism for synthesis of phthalimides from 1,4-dimethoxyphthalazines.

Chung and co-workers disclosed an oxidative approach for the synthesis of phthalimides *via* an unusual ring formation in 1,4-dimethoxyphthalazines (Scheme 43).^72^ For this purpose, an electrophilic chlorinating reagent, such as trichloroisocyanuric acid (TCICA) was used to chlorinate the N-atom of substrate 64. The conversion of the 6-membered ring substrate to the 5-membered ring product was initiated by the first *N*-chlorination of 64 by TCICA, followed by the second *N*-chlorination to form the cationic intermediate A. Then, an intramolecular C–N bond formation in A led to the strained bicyclic intermediate B, which was readily opened by a nucleophile in the reaction mixture to obtain a more stable intermediate C. Next, C was transformed into intermediate D and then into product 65 with the loss of an activated methyl group (Scheme 44). DFT calculations confirmed the formation of transition states and the tentative mechanism.

**Scheme 45:**
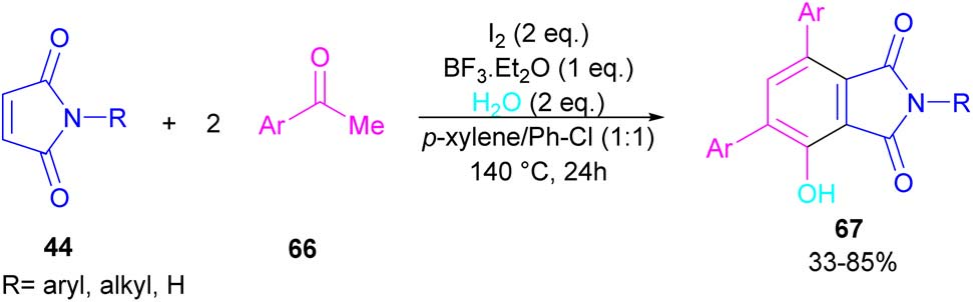
The I_2_-mediated three-component reaction of maleimides, acetophenones and H_2_O.

**Scheme 46:**
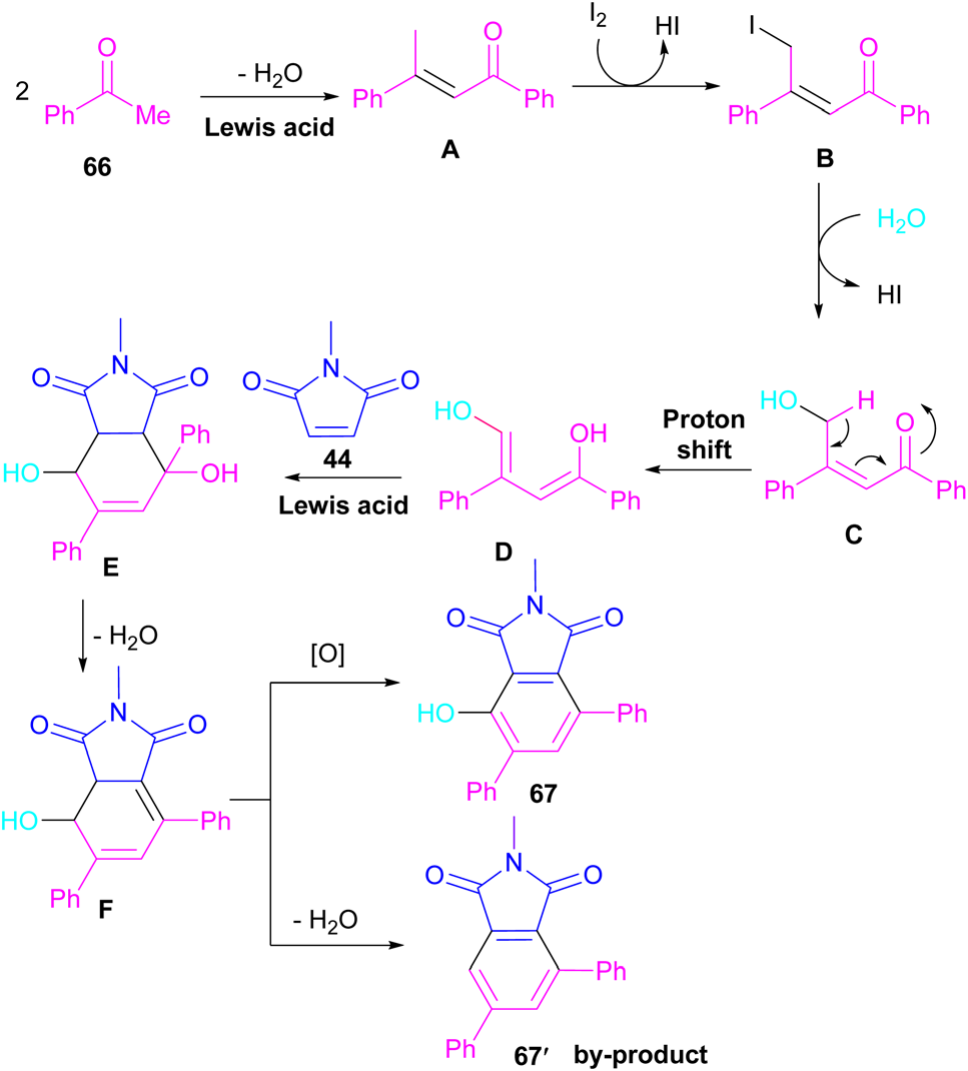
Possible mechanism for I_2_-mediated three-component reaction of maleimides, acetophenones and H_2_O.

A novel library of highly functionalized phthalimides 67 was constructed by Deng and co-workers in 2023 (Scheme 45).^73^ In this strategy, maleimides 44 and acetophenones 66 were used as starting materials and H_2_O acted as an oxygen source. By the H_2_O^18^ isotope labelling experiment, the authors could prove that the oxygen of phenolic in the product originated from H_2_O. As outlined in Scheme 46, the reaction mechanism involved the initial self-condensation of acetophenones 66 in the presence of Lewis acid. Then, dypnone A was transformed to iododypnone B in the presence of I_2_. The nucleophilic attack of H_2_O to B yielded intermediate C, which underwent an H-shift to generate intermediate D. After that, (4 + 2)-cycloaddition between D and 44 gave intermediate E, which dehydrated and oxidized to form product 67. Further dehydration of F could also lead to by-product 67′.

## 3. *N*-Functionalizations of phthalimides

### 3.1. *N*-Arylation

**Scheme 47:**
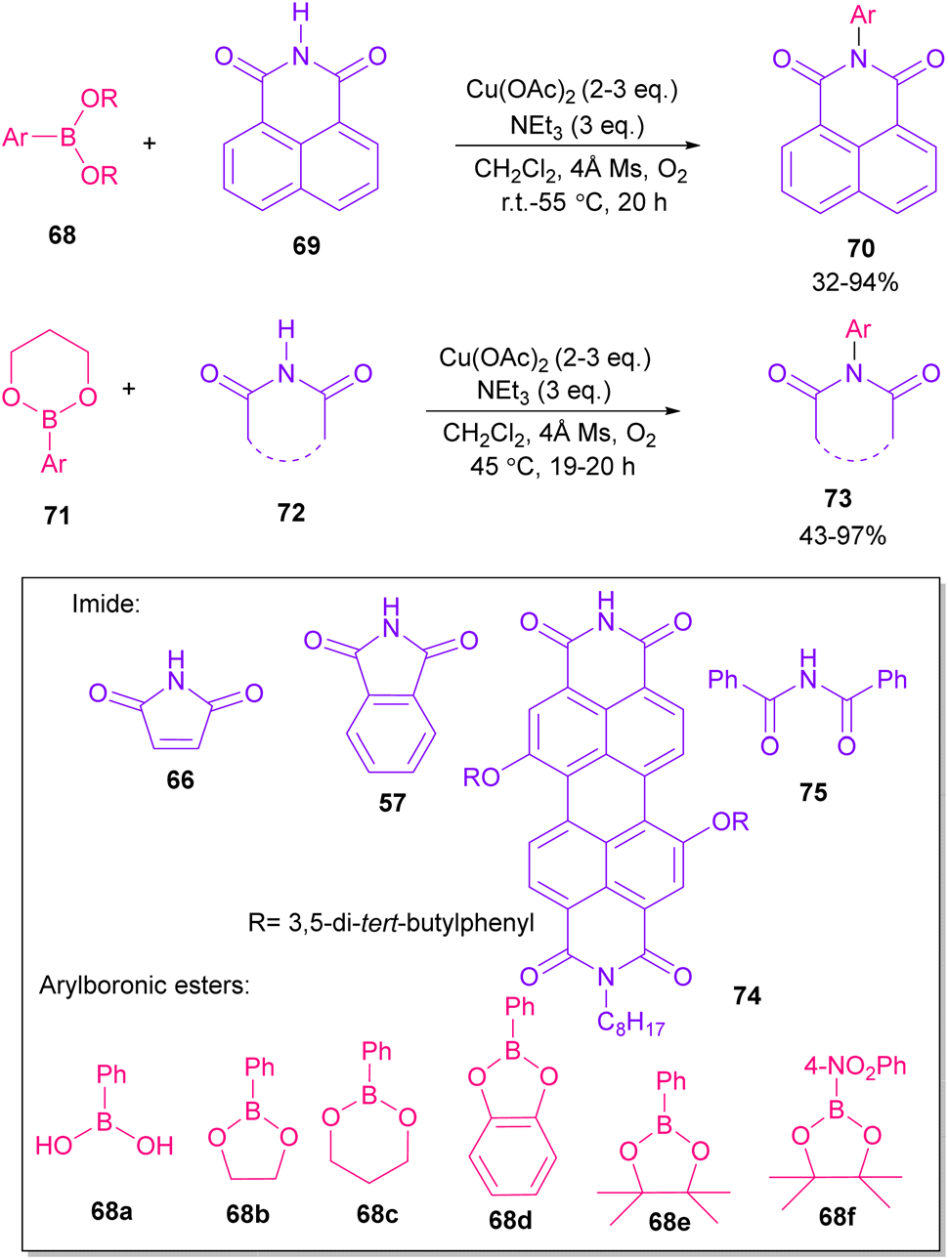
Cu-promoted *N*-arylations of cyclic imides.

**Scheme 48:**
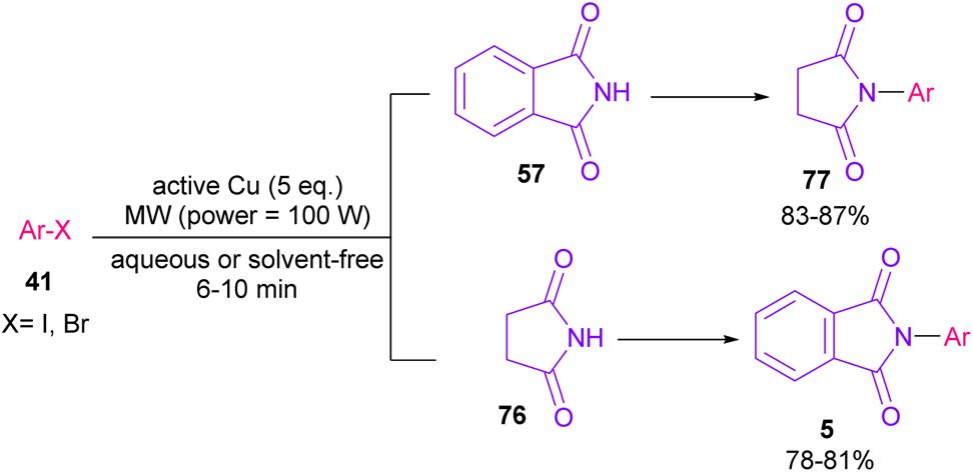
Active Cu-promoted *N*-arylation of imides.

In 2005, *N*-arylation of several cyclic imides, including naphthalimide 69, maleimide 44, phthalimide 57, and perylenebis(imide) 74 was performed by Wasielewski and co-workers (Scheme 47).^74^ A wide range of arylboronic esters were compatible in this work. However, dibenzamide 75 and phenyl boron pinacolate 68 did not participate in this arylation. After a while, an active copper was used for *N*-arylation of various amines, amides, β-lactams and imides under microwave irradiation (Scheme 48).^75^ Phthalimide 5 and maleimide 44 as the imide substrates led to the *N*-arylated products in high yields. This method has advantages of short reaction time, high yields and the performance of the reaction in aqueous media or under solvent-free conditions. However, the products were obtained in lower yields in a solvent-free system.

**Scheme 49:**
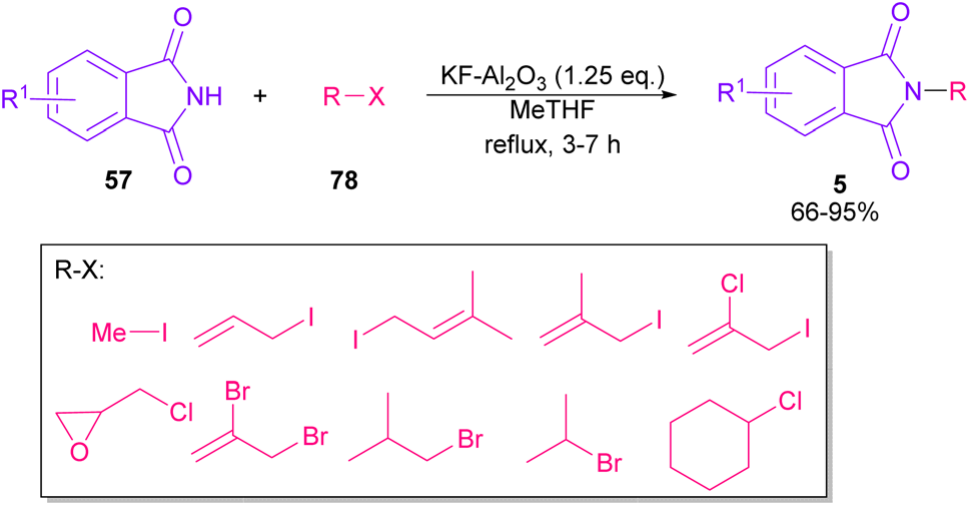
*N*-Functionalization of phthalimides with electrophilic reagents.

**Scheme 50:**
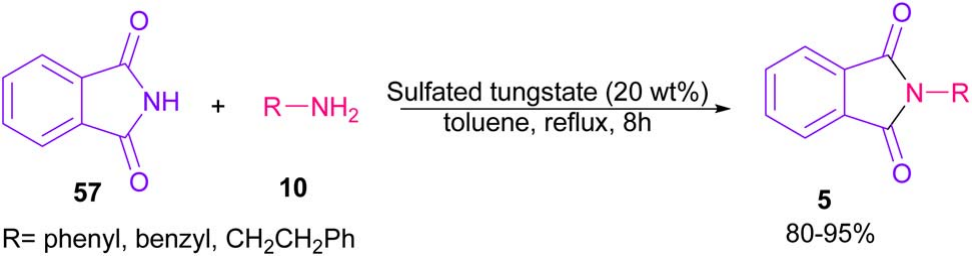
Sulfated tungstate-catalyzed transamidation of carboxamides with amines.

In 2010, KF supported by alumina was utilized for *N*-functionalization of phthalimides 57 (Scheme 49).^76^ Strong basicity of KF-Al_2_O_3_ can efficiently abstract the N–H imide and increase the nucleophilicity of the nitrogen of phthalimide. Various electrophilic reagents, such as allyl halides, secondary alkyl halides, epichlorohydrin, and methyl iodide well participated in the reaction with phthalimides. Among them, primary electrophiles gave partially higher yields compared to secondary electrophiles possibly due to the steric hindrance. In addition, the non-formation of the elimination byproduct indicates the efficiency and chemoselectivity of this transformation. In 2013, transamidation of NH-phthalimides 57 with amines 10 was extended in the presence of sulfated tungstate as a heterogeneous catalyst (Scheme 50).^77^ In addition to NH-phthalimides, various amides, such as formamide, benzamide, and acetamide led to the corresponding *N*-substituted amides.

**Scheme 51:**
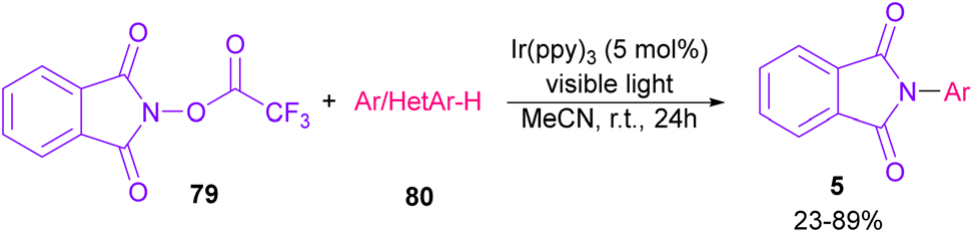
Ir-catalyzed *N*-arylation of *N*-acyloxyphthalimides.

**Scheme 52:**
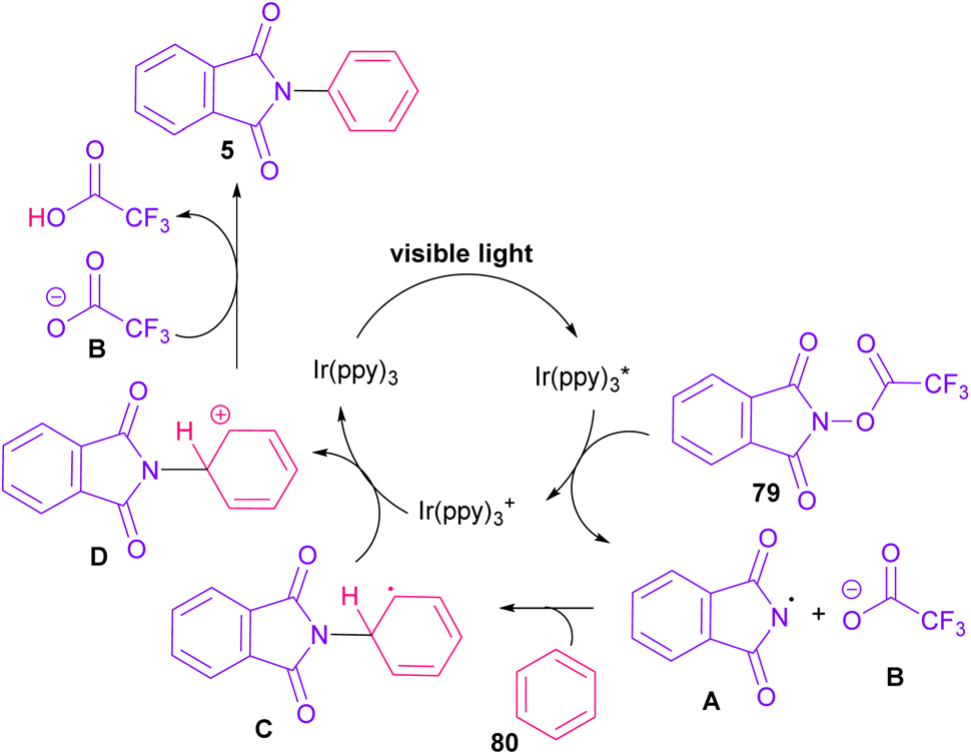
Catalytic cycle for Ir-catalyzed *N*-arylation of *N*-acyloxyphthalimides.

**Scheme 53:**
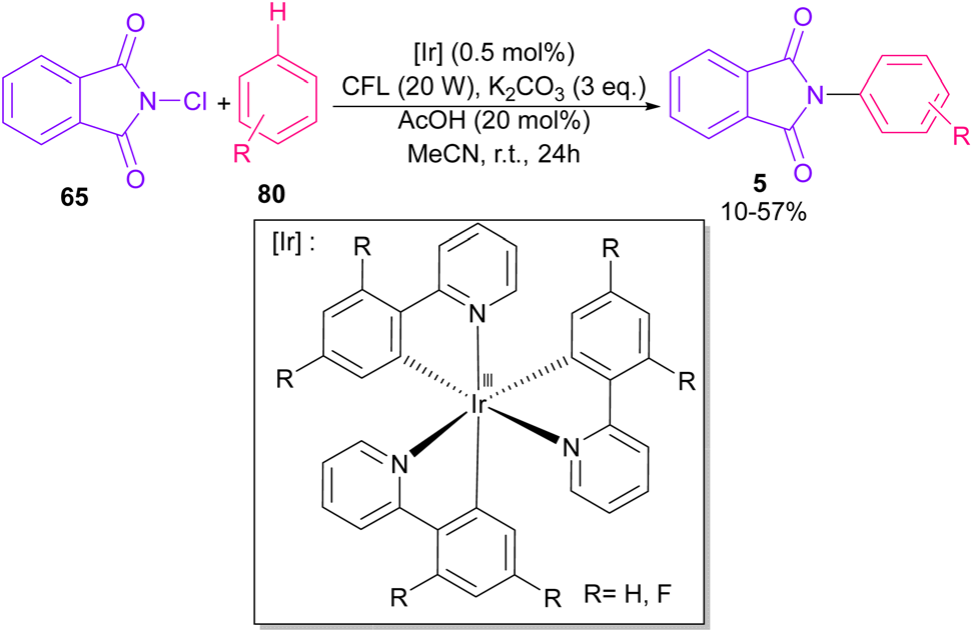
Ir-catalyzed *N*-arylation of *N*-chlorophthalimides.

**Scheme 54:**
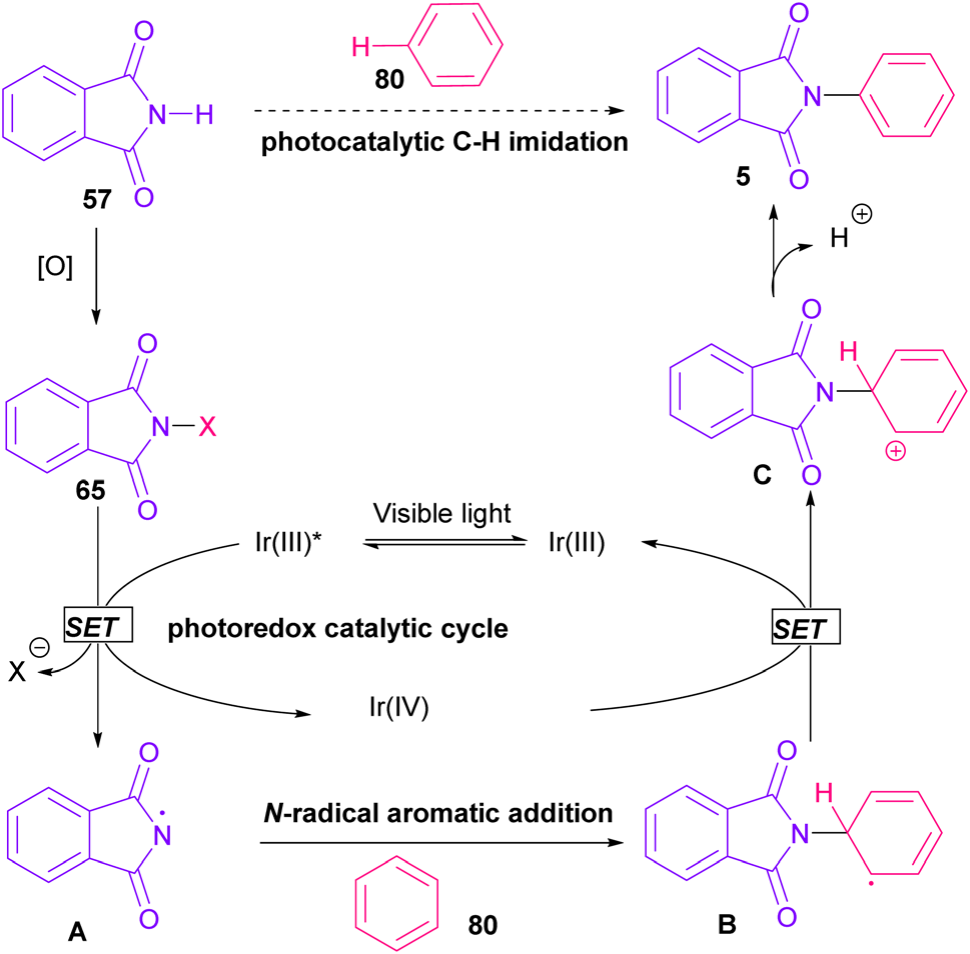
Possible pathway for Ir-catalyzed *N*-arylation of *N*-chlorophthalimides.

In 2014, Sanford and co-workers employed an iridium photocatalyst for the arylation of *N*-acyloxyphthalimides by using aromatic and heretoaromatic precursors (Scheme 51).^78^ Preliminary mechanistic investigation revealed the necessity of visible light for the reaction progress and the involvement of a radical route. According to the mechanism in Scheme 52, photo-excited state 
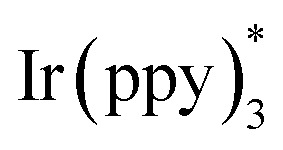
 was generated from ground state Ir(ppy)_3_ under visible light irradiation, followed by a single electron transfer to 79, resulting in the *N*-centered phthalimidyl radical A, OTf^−^B, and Ir(ppy)_3_^+^. After that, the attack of the radical A to arene 80 led to a neutral radical intermediate C, which was oxidized by Ir(ppy)_3_^+^ to regenerate the cationic intermediate D and Ir(ppy)_3_. Finally, OTf^−^B abstracted a proton from D to liberate product 5 and HOTf. In the same year, another iridium photocatalytic system was extended for the arylation of 2-chlorophthalimides using various arenes (Scheme 53).^79^ In this method, irradiation of Ir(iii) led to photo-excited state Ir(iii)*, which transferred an electron to the N–X bond in substrate 65 to cleavage the N–X bond towards the N-radical intermediate A. By the addition of A to arene 80, the C-radical B was obtained, which underwent an SET reaction with Ir(iv) to afford the carbocation (Wheland intermediate) C with the regeneration of Ir(iii). Finally, OTf^−^ abstracted a proton from the cation C to furnish product 5 and HOTf (Scheme 54).

**Scheme 55:**
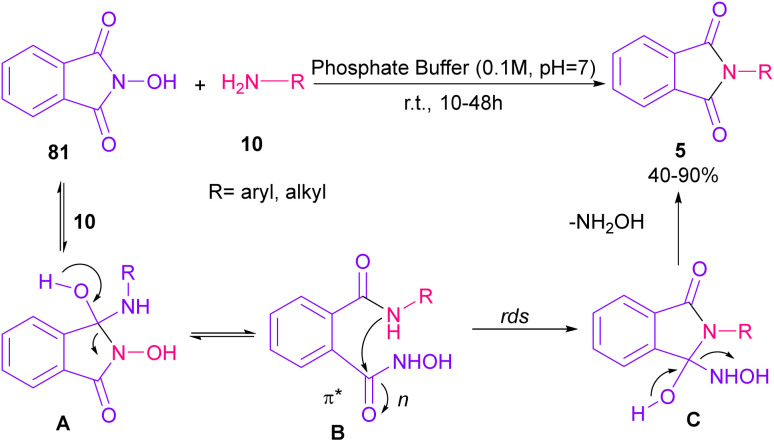
Phthalimidation of phthalic anhydride by using amines.

Phthalimidation of *N*-hydroxyphthalimide 81 using aromatic and aliphatic amines 10 in the presence of a solution of phosphate buffer resulted in the formation of phthalimide derivatives 5 (Scheme 55).^80^ The control of reactivity of the amphoteric intermediate resulted in good chemoselectivity. In general, the reaction proceeded in a one-pot two-stage transformation, involving the ring opening of 81, followed by the nucleophilic addition of amine 10 to form intermediate A, which was converted to intermediate B. Then, intramolecular nucleophilic attack on the electrophilic carbonyl resulted in intermediate C in the rate-determining step. The hydroxyl group can control the electrophilicity of the carbonyl group by regulating n to π* contribution. The removal of NH_2_OH afforded product 5. Finally, the utility of products was investigated in chemoselective and regioselective labelling of a protein.

**Scheme 56:**
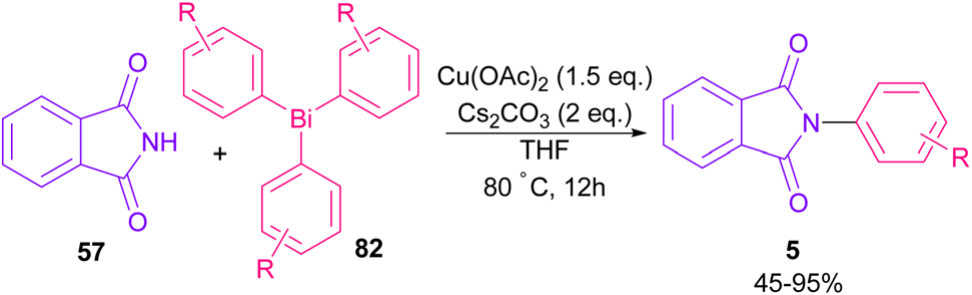
Cu-catalyzed *N*-arylation of phthalimides with triaryl bismuth.

**Scheme 57:**
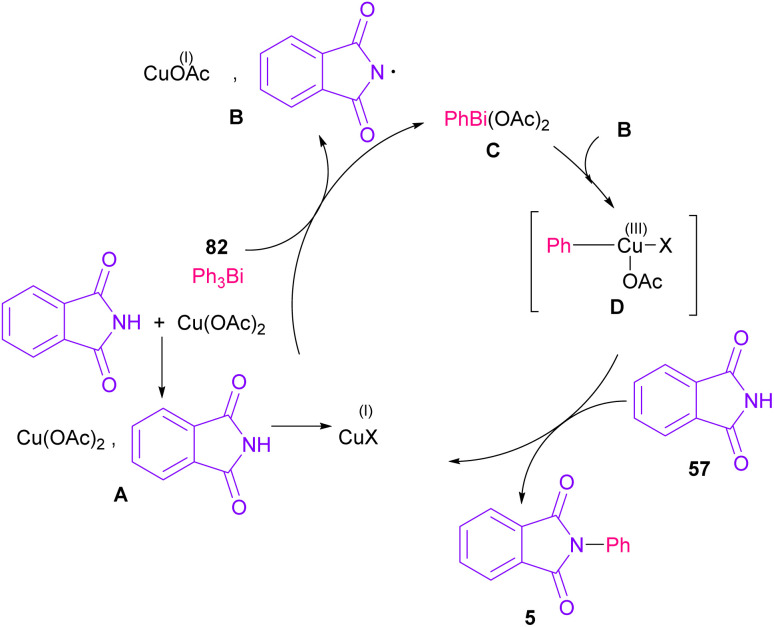
Possible mechanism for Cu-catalyzed *N*-arylation of phthalimides with triaryl bismuth.

The arylation of phthalimides 57 using triaryl bismuth 82 can be carried out in the presence of Cu(OAc)_2_ as a catalyst (Scheme 56).^81^ The steric hindrance of the triaryl bismuth has an important role in the transmetallation from bismuth to copper. *Ortho*-methyl triphenyl bismuth had a negative effect on the reaction. In addition, the nature of functional groups on the aryl ring of triaryl bismuth has a significant effect on the reaction progress. For example, the halo substituents at the aryl ring decreased the reactivity, whereas electron-donating groups, such as Me and OMe have a positive effect on the reaction. The mechanism was initiated by the conversion of Cu(ii) to Cu(i), which was then incorporated in the oxidative addition with Ar_3_Bi 82 to give Cu(i)OAc and ArBi(OAc)_2_C. In the next step, C reacted with B to generate the Cu(iii) intermediate D. The coupling of the aryl moiety of D with phthalimide 57 led to the *N*-arylated phthalimide 5. The generated Cu(i)X could start the next catalytic cycle (Scheme 57). In the meantime, another copper catalytic arylation of phthalimides was carried out.^82^ In this method, a series of aryldiazonium tetrafluoroborate was used as an arylating reagent, which could be decomposed to an aryl radical and N_2_ under Cu catalysis.

**Scheme 58:**
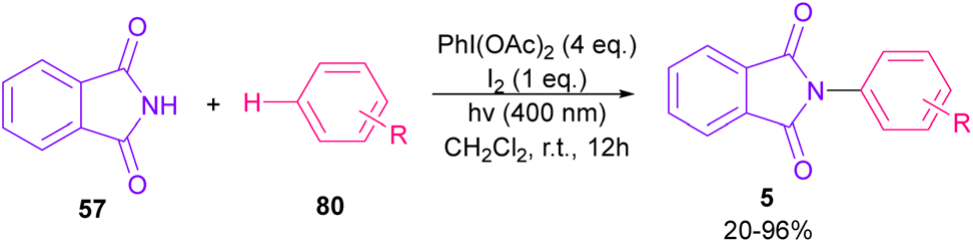
Visible light-mediated *N*-arylation of phthalimides with arenes.

**Scheme 59:**
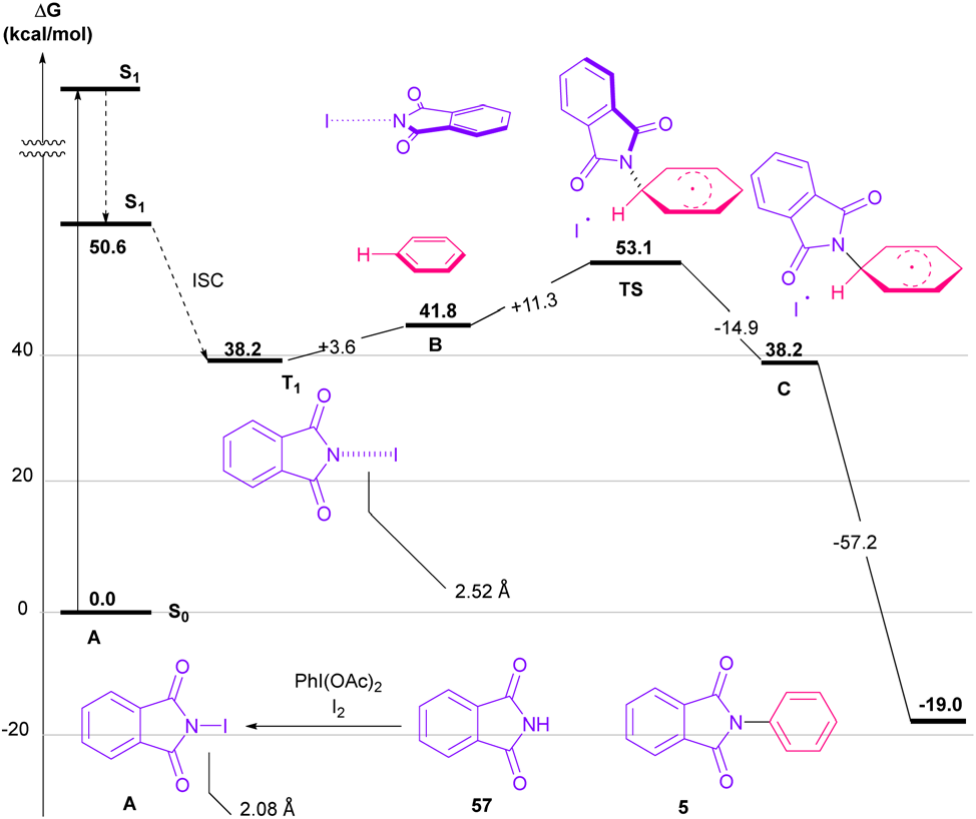
Proposed mechanism using DFT calculations for visible light-mediated *N*-arylation of phthalimides with arenes.

Nemoto *et al.* developed a visible light strategy for the preparation of the *N*-arylated phthalimides 5 (Scheme 58).^83^ The arylation proceeded through the photolysis of N-iodophthalimide intermediate A, which was generated from the iodination of phthalimide by PhI(OAc)_2_/I_2_. According to the DFT calculations, the photolysis of *N*-iodophthalimide A in the presence of visible light proceeded *via* transition state T1. In this TS, the length of the N–I bond in the triplet excited state is longer than the ground state, which showed the easy cleavage of the N–I bond in the triplet excited state. The formation of the *N*-arylated phthalimidyl radical occurred with a low activation energy (11.3 kcal mol^−1^). Finally, the aromatization of the radical intermediate C to *N*-aryl phthalimide 5 was carried out thermodynamically (Scheme 59). This protocol provided a metal-, and photocatalyst-free synthesis of a wide range of phthalimide derivatives up to excellent yield.

### 3.2. *N*-Alkenylation

**Scheme 60:**
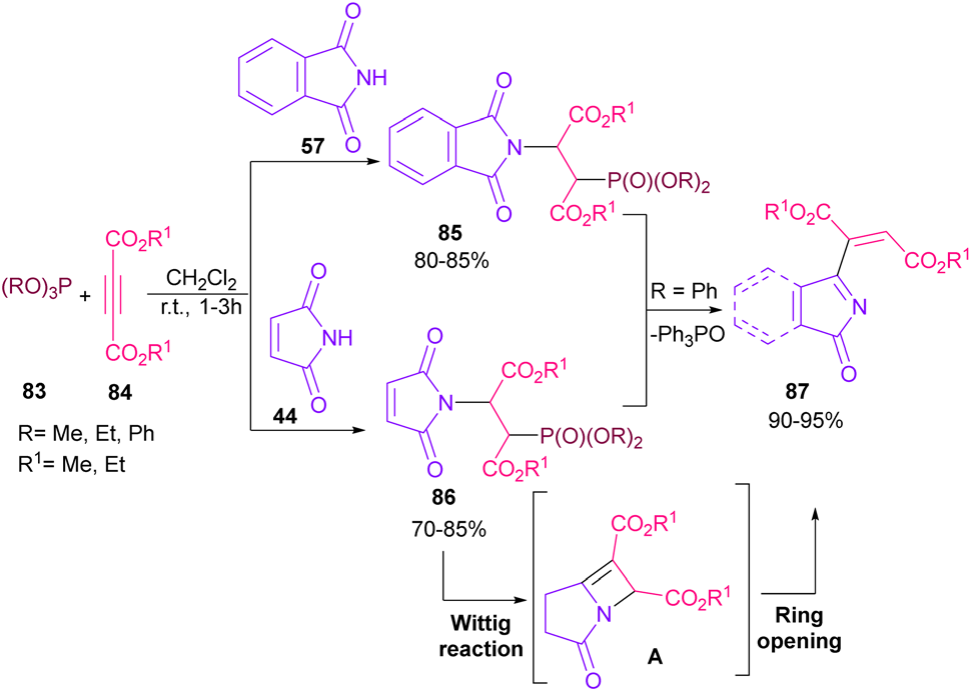
*N*-Functionalization of phthalimides with electrophilic reagents.

In 2021, functionalization of phthalimide 57 and maleimide 44 through a three-component reaction, including phthalimide/maleimide 57, 44, dialkyl acetylene dicarboxylates 84 and trialkyl/aryl phosphites 83 was carried out under catalyst-free conditions (Scheme 60).^84^ When triphenylphosphine was used as reactant, *N*-substituted phthalimide/maleimide 85, 86 could be transformed into functionalized 2*H*-pyrrol-2-one derivatives 87*via* an intramolecular Wittig reaction and subsequent electrophilic ring opening step.

**Scheme 61:**
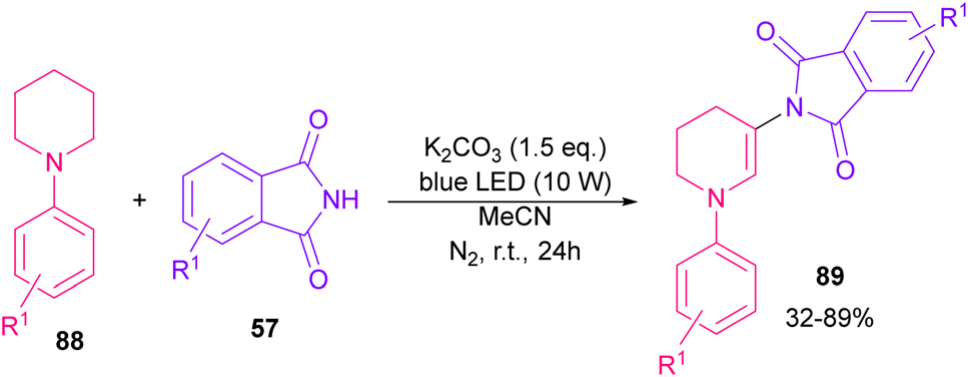
Visible light-mediated reaction of *N*-phenylpiperidine and phthalimide.

**Scheme 62:**
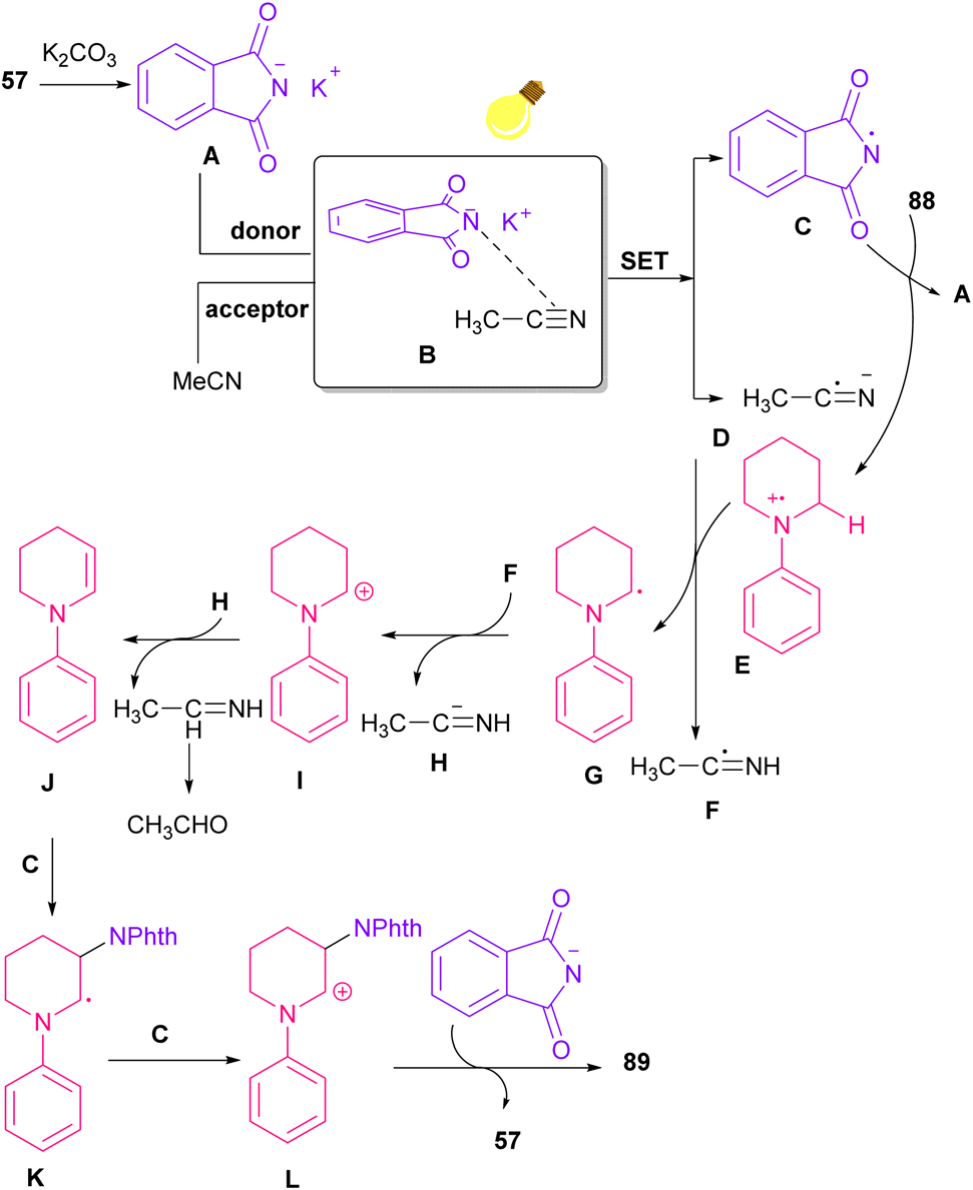
Possible mechanism for the visible light-mediated reaction of *N*-phenylpiperidine and phthalimide.

The reaction of phthalimides and *N*-phenylpiperidine was carried out under visible light conditions (Scheme 61).^85^ This method has the advantages of the absence of metal or photocatalyst, mild reaction conditions, and broad substrate tolerance. The reaction started with the deprotonation of phthalimide by K_2_CO_3_ to obtain salt A. A donor–acceptor complex B was formed by the interaction of MeCN with potassium phthalimidate A, which underwent a single electron transfer (SET) by visible light irradiation to produce a phthalimide radical (PhthN˙) C and a radical anion D. Then, C abstracted an electron from *N*-phenylpiperidine 88 to form a radical cation E and regenerate A. Next, D abstracted a proton from E to give another radical F and G. Another SET reaction between F and G gave a cation I, which underwent deprotonation to yield an enamine J. In this step, C could attack the β-position of enamine J to render a radical K, followed by another SET process with B to form a cation L. Finally, the deprotonation of L by anion phthalimide furnished product 89 (Scheme 62).

**Scheme 63:**
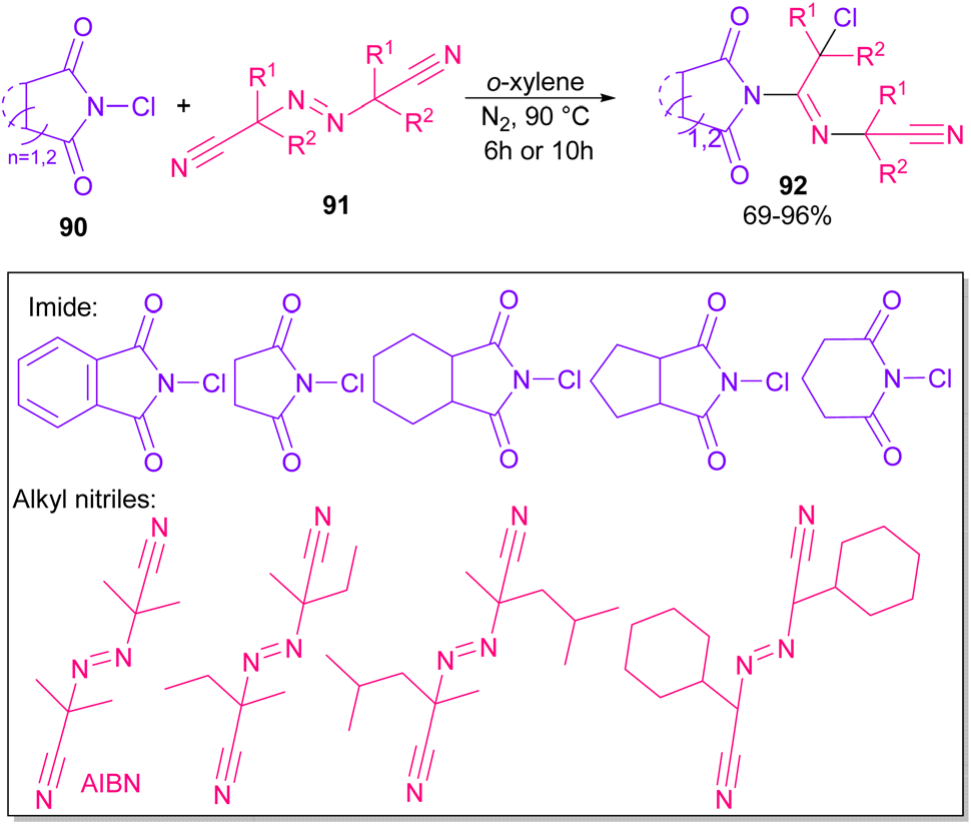
Reaction of *N*-chlorimides with azonitriles.

**Scheme 64:**
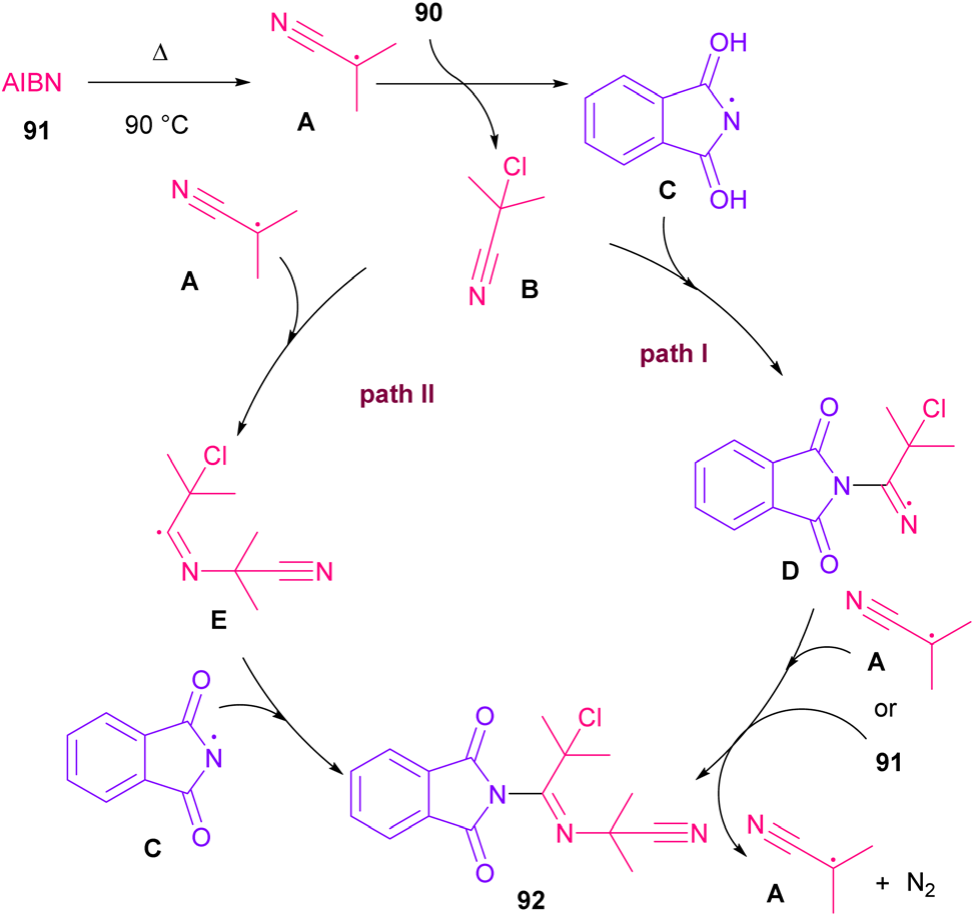
Possible mechanism for the reaction of *N*-chlorimides with azonitriles.

Azobis(isobutyronitrile) (AIBN) can serve as a radical precursor to incorporate in functionalization of imides (Scheme 63).^86^ Various imides such as *N*-chlorophthalimide, *N*-chlorosuccinimide, *N*-chloroglutarimide and other succinimide derivatives were well reacted with azonitriles to construct *N*-functionalized imide derivatives. The reaction involved the formation of the cyanoisopropyl radical A under thermal conditions, which quenched a chlorine radical from phthalimide A to obtain the nitrogen radical C and chloro nitrile B. According to DFT calculations, the authors suggested two pathways for intermediate B to give product 92. In path I, C was added to B to produce the imine radical D, which then coupled with A or AIBN to yield product 3aa. In path II, A was attacked by the nitrogen of B to generate the carbon radical E, which underwent subsequent coupling with C to deliver product 92. DFT calculations showed that the formation of intermediate D is more favored due to the lower energy barrier (Scheme 64).

**Scheme 65:**
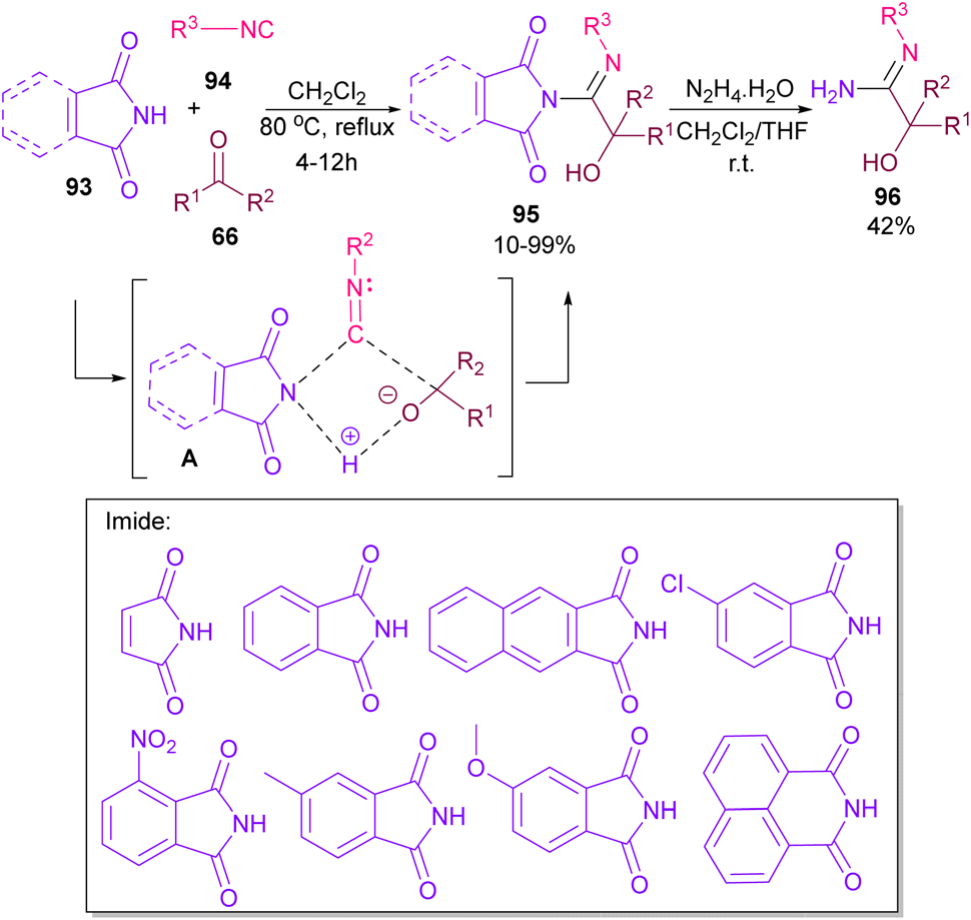
Three-component reaction of phthalimides, isocyanides and ketones.

Very recently, Dömling and co-workers explored a metal-free methodology for the functionalization of phthalimides using isocyanides and ketones as coupling partners (Scheme 65).^87^ The method involved a Passerini reaction, in which NH of phthalimide acted as an acid precursor and enabled activation of the carbonyl moiety of ketone 66. The activated carbonyl underwent the attack of isocyanide 94. Meantime, the N-atom of phthalimide interacted with the C-atom of isocyanide to form the cyclic intermediate A. Finally, alcohol 95 was formed by a proton shift from phthalimide to the oxygen of ketone. Various phthalimide derivatives as well as maleimide could be served as an acid reactant in this Passerini reaction. Moreover, a further transformation of phthalimide 95 in the presence of hydrazine led to amidine 96.

## 4. Conclusions

As shown in this review, various transition metals can catalyze the synthesis of phthalimides from *ortho*-dihaloarenes, *ortho*-haloacids/esters/benzamides, cyclic ketones, cyclic amines, maleimides, *etc.* Also, phthalimides can be obtained from phthalic anhydrides, aldehydes, phthalic acids, *ortho*-formylbenzoic acids, *ortho*-dicyanoarenes, cyclic ketones and benzamides under metal-free reactions. Although reactions using metal catalysts resulted in higher efficiency, the use of organocatalysts and visible light irradiation displayed a reliable and promising system for phthalimide synthesis.

Nevertheless significant achievements in the synthesis of phthalimide cores, the construction of highly functionalized phthalimides is still of great challenge for the synthetic community.

Other issues in this field are the use of CO gas and noble metal catalysts in the phthalimide synthesis that are better replaced with other green synthetic methods. Finding safe and sustainable C1 precursors and developments in catalyst-free photochemical, and electrochemical systems seems to be a good alternative in this field. Also, the use of chiral organocatalysts in the synthesis of enantioselective phthalimides is still underexplored.

In addition, the acidic nature of the imide moiety in phthalimide allows it to be incorporated into hydrogen bonding interaction, leading to good solubility in polar solvents. The formation of stable complexes through the chelation with metals makes it an invaluable starting material or intermediate for the preparation of various types of bioactive molecules, such as alkaloids and pharmacophores. So far, it has been observed that phthalimide and its analogues have shown similar or even better biological effects than known pharmaceutical products, so their biological activity is one of the important topics of biomedical research.

## Data availability

All data of this manuscript are available.

## Conflicts of interest

There are no conflicts to declare.
